# Fruit and vegetable intake and the risk of non-alcoholic fatty liver disease: a meta-analysis of observational studies

**DOI:** 10.3389/fnut.2024.1398184

**Published:** 2024-06-21

**Authors:** Rui Wang, Ruijuan Yan, Junzhe Jiao, Feilong Li, Haibo Zhang, Zhanjie Chang, Hailiang Wei, Shuguang Yan, Jingtao Li

**Affiliations:** ^1^The First Clinical Medical College, Shaanxi University of Chinese Medicine, Xianyang, China; ^2^Department of Hepatology, The Affiliated Hospital of Shaanxi University of Chinese Medicine, Xianyang, China; ^3^School of Integrated Traditional Chinese and Western Medicine, Southwest Medical University, Luzhou, China; ^4^Advanced Instituted of Medicine Sciences, Dalian Medical University, Dalian, China; ^5^Department of General Surgery, The Affiliated Hospital of Shaanxi University of Chinese Medicine, Xianyang, China

**Keywords:** vegetable, fruit, non-alcoholic fatty liver disease, diet, meta-analysis

## Abstract

**Purpose:**

This systematic review and meta-analysis of clinical observational studies aims to clarify the correlation between the intake levels of fruits and vegetables and non-alcoholic fatty liver disease (NAFLD).

**Materials and methods:**

PubMed, Embase, Web of Science, and the Cochrane Library were searched for studies on the association between vegetable or fruit intake with the risk of NAFLD from the foundation of each database up until September 2023. The relative risk (OR) and the 95% confidence interval (CI) were pooled for both the highest and lowest consumption levels of vegetables and fruits to explore their association with the incidence of NAFLD.

**Results:**

The meta-analysis encompassed 11 studies with a total of 493,682 patients. A higher consumption of vegetables (OR = 0.78, 95% CI = 0.67–0.91) and fruits (OR = 0.88, 95% CI = 0.83–0.93) was found to have a negative correlation with the risk of NAFLD, denoting an inverse association. This correlation, however, varied among different ethnic groups and gender.

**Conclusions:**

Our results indicate that increased consumption of vegetables and fruits is associated with a reduced likelihood of developing NAFLD.

**Systematic review registration:**

https://www.crd.york.ac.uk/PROSPERO/#searchadvanced, identifier: CRD42023460430.

## 1 Introduction

NAFLD, predominantly caused by metabolic syndrome, is closely associated with obesity, insulin resistance and hyperlipidemia (1). Over the past four decades, the incidence of NAFLD has steadily increased (2, 3), currently affecting 25% of the global adult population (3). The disease prevalence is about 30% in Asia (4), the US, and South America, 24% in Europe, and 13% in Africa (2, 5–7), making it the most widespread chronic liver disease worldwide (2). An estimated 20% of non-alcoholic steatohepatitis (NASH) patients progress to cirrhosis, and the risk of hepatocellular carcinoma in NASH patients surged 7.7·times between 2002 and 2016, from 2.1 to 16.2% (8). In the US, it is expected that NASH medical expenses per patient will jump from $3,636 to $6,968 between 2020 and 2039 (9). Similarly, in Japan, the annual healthcare cost for NASH ranged from 322,206 to 340,399 yen per patient between 2011 and 2017 (10), imposing a substantial economic burden. Therefore, attention must be focused on early disease detection in primary healthcare settings.

Obesity, over nutrition, a high-calorie diet, and a sedentary lifestyle contribute to the accumulation of liver fat (11, 12), and are crucial risk factors for NAFLD (13). The regulatory mechanism of NAFLD is connected with metabolism, heredity, intestinal microorganisms, and other factors (11, 14, 15). At present, the management of NAFLD is centered around reducing insulin resistance and limiting oxidative stress (13). Treatment strategies are founded on lifestyle management, such as modifying diet and increasing physical activity, with the intent of controlling weight and managing risk factors pertinent to metabolic syndrome (16, 17). Consequently, adhering to a balanced diet and maintaining a healthy lifestyle have become pivotal in treating and delaying the progression of this disease (18, 19).

Fruits and vegetables are plant-based foods, rich in dietary fiber, which can help maintain the balance of intestinal flora, reduce inflammation, and decrease fat accumulation in the liver. Moreover, fruits and vegetables are abundant in antioxidants that neutralize free radicals and diminish oxidative stress damage to the liver. The antioxidants (20) and anti-inflammatory compounds (21) in fruits and vegetables enhance insulin sensitivity, accelerate beta-oxidation, and inhibit new fat production (22). As a result, it has been hypothesized that an intake of fruits and vegetables correlates with a lower prevalence of NAFLD (23). However, the relationship between fruit and vegetable intake and NAFLD risk remains a subject of debate. Several observational studies suggest that higher dietary vegetable intake is associated with a lower NAFLD risk (24, 25). Yet, some research indicates that there is no such relationship (23, 26). A similar controversy exists in regard to the correlation between fruit intake and the risk of NAFLD (26–31). Even though the role of vegetable and fruit intake in NAFLD has drawn considerable public attention, no meta-analyses have demonstrated a correlation between vegetable and fruit consumption and NAFLD risk. Therefore, we undertook this meta-analysis to summarize the results of observational studies regarding the association between vegetable and fruit consumption and the risk of NAFLD.

## 2 Materials and methods

This systematic review and meta-analysis statement followed Preferred Reporting Items for Systematic Reviews and Meta-Analyses (PRISMA), and the protocol was registered with **PROSPERO (ID: CRD42023460430)**.

### 2.1 Study strategy

The researchers scoured Pubmed, Embase, Web of Science, and the Cochrane Library for studies on the correlation between vegetable or fruit consumption with the risk of NAFLD from the inception of these databases to September 2023. Key search terms included non-alcoholic Fatty Liver Disease, fruit^*^, vegetable^*^, among others. Detailed search strategies are presented in the Supplementary material. The Endnote software (X20 version) was utilized to eliminate duplicate documents fetched from each database, and the remaining potentially eligible documents were manually screened (Appendix 1).

### 2.2 Inclusion and exclusion criteria

Two researchers checked the titles and abstracts to select studies that met the inclusion and exclusion criteria. The full texts of these studies were examined to choose eligible studies. In instances where consensus on eligibility could not be reached, a third reviewer was engaged for discussion.

Inclusion criteria were as follows: (1) studies assessing the correlation between varying levels of vegetable and fruit consumption, and the risk of NAFLD; (2) studies that provide relative risk, odds ratio, hazard ratio, and their corresponding 95% confidence intervals; (3) observational studies such as cross-sectional studies, case-control studies, and cohort studies.

Papers were ineligible for inclusion using the following criteria: (1) duplicate papers; (2) irrelevance to the subject matter (irrelevant disease and observation indicators); (3) meta-analysis, reviews, letters, conference abstracts, case reports, guidelines, etc.; (4) animal experiments.

### 2.3 Data extraction

Two reviewers extracted basic information from the articles finally included. This information comprised the first author, publication year, country, study type, sample size, age of study population, sex ratio, follow-up time, disease diagnosis method, intake assessment method, model adjustment factors, and the relative risk RR (OR, HR) associated with the highest and lowest fruit and/or vegetable intake along with their corresponding 95% confidence intervals. Where possible, the maximally adjusted RR, OR, or HR ratio and 95% CI were extracted. Any disagreements during the review process, if any, were resolved by discussion or, if necessary, consultation with a third reviewer.

### 2.4 Quality assessment

The assessment of bias and quality of the included studies was performed independently by two reviewers, with discrepancies resolved by a third reviewer. The quality assessment adhered to our published protocols. The quality of case-control studies was evaluated using the Newcastle-Ottawa Scale (NOS) and categorized into high quality (score 7–9), medium quality (score 4–6), and low quality (score 0–3). The quality of cross-sectional studies was assessed using the AHRQ scale from the U.S. Agency for Healthcare Research and Quality, and these cross-sectional studies were assessed as low quality (score 0–3), medium quality (score 4–7), or high quality (score 8–11).

### 2.5 Statistical analysis

In this study, Stata (version 15.0) was utilized to gather and summarize the OR and its corresponding 95% confidence interval, and to develop a forest map. Heterogeneity was evaluated by the *Q*-test and *I*-square test. A random effects model was employed when I-squared was ≥50% and *p* < 0.1; under other circumstances, a fixed effects model was used. In the presence of high heterogeneity, we conducted subgroup (study type, continent, and intake assessment questionnaire) and stratified analyses to explore heterogeneity sources. Sensitivity analysis was performed by observing the results' stability after sequentially eliminating each article. The potential risk of publication bias was assessed by examining funnel plots. When dealing with ≧10 articles, publication bias was evaluated using Egger's test and Begg's test. If publication bias was present, further evaluation was conducted using the “trim-and-fill” method. A bilateral *P* < 0.05 indicates a notable distinction.

## 3 Results

### 3.1 Literature search and selection

**Figure 1:**
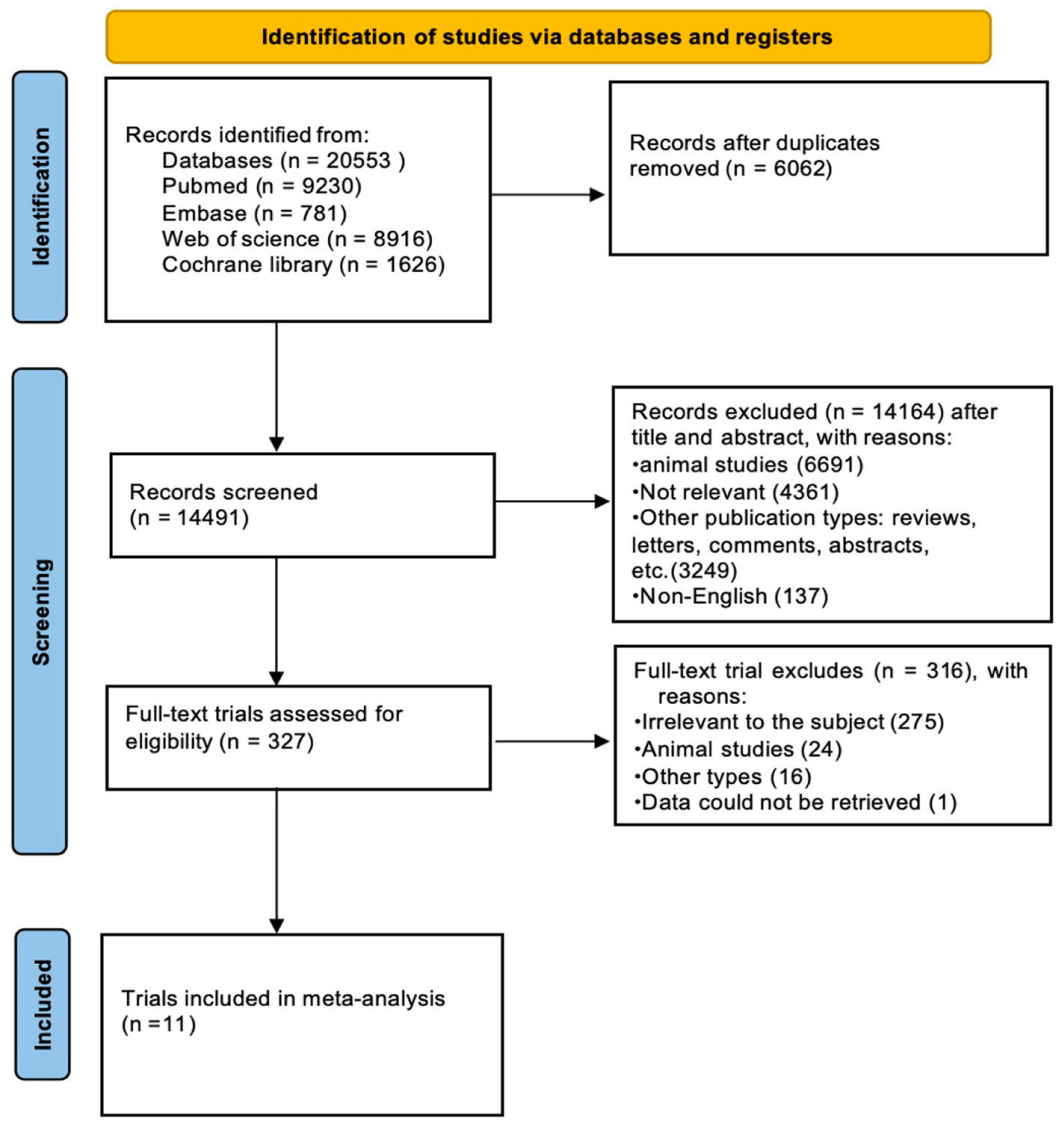
Flow diagram of study selection process.

Papers related to the association between vegetable and/or fruit intake and the risk of NAFLD were searched from Pubmed (9,230 articles), Embase (781 articles), Web of Science (8,916 articles), and Cochrane Library (1,626 articles) from the inception of these databases until September 2023. After utilizing EndNote(X20) for automatic duplication removal, 14,491 related publications remained. Following a manual check for duplicates, 327 articles were left. These were then screened by their titles and abstracts according to inclusion and exclusion criteria. After a full-text review, only 11 papers were included. The literature screening process is illustrated in Figure 1.

### 3.2 Study characteristics

The 11 included studies comprised six cohort studies and five case-control studies. Of the chosen studies, eight were conducted in Asian countries, specifically China (four studies), Iran (two studies), South Korea (one study), and Japan (one study). Additionally, two studies were conducted in Europe (one in Italy and one in the United Kingdom), and one study was conducted in North America (the United States). There was a total of 493,682 participants, consisting of 221,779 males and 271,901 females, whose ages ranged from 18 to 79 years. All included studies were deemed to be of high quality considering their AHRQ and NOS scores (Tables 1, 2). In the included studies, three intake assessment questionnaires were used, including Food Frequency Questionnaire (FFQ) used in nine studies, BDHQ in one study, and Food Diary in one study. The attributes of the included studies are detailed in Table 3.

**Table 1:** Quality assessment of six case-control studies.

**References**	**Selection**				**Comparability**	**Exposure**			**Overall score**
	**Is the case definition adequate?**	**Representativeness of the cases**	**Selection of controls**	**Definition of Controls**	**Comparability of cohorts on the basis of the design or analysis**	**Ascertainment of exposure**	**Same method of ascertainment for cases and controls**	**Non-response rate**	
Emamat et al. (24)	1	1	1	1	2	-	1	-	7
Giraldi et al. (32)	1	1	1	1	2	-	1	-	7
Noureddin et al. (29)	1	1	1	1	2	-	1	1	8
Tutunchi et al. (30)	1	1	1	1	2	-	1	1	8
Guo et al. (26)	1	1	1	1	2	-	1	1	8

**Table 2:** Quality assessment of six cross-sectional studies.

	**Chan et al. (27)**	**Liu et al. (28)**	**Tajima et al. (23)**	**Kim and Shin (25)**	**Li et al. (33)**	**Du et al. (31)**
Define the source of information (survey, record review)	1	1	1	1	1	1
List inclusion and exclusion criteria for exposed and unexposed subjects (cases and controls) or refer to previous publications.	1	1	1	1	1	1
Indicate time period used for identifying patients.	1	1	1	1	1	1
Indicate whether or not subjects were consecutive if not population-based.	1	1	1	1	1	1
Indicate if evaluators of subjective components of study were masked to other aspects of the status of the participants.	1	1	1	1	1	1
Describe any assessments undertaken for quality assurance purposes (e.g., test/retest of primary outcome measurements).	1	1	1	1	1	1
Explain any patient exclusions from analysis.	0	0	0	0	0	0
Describe how confounding was assessed and/or controlled.	1	1	1	1	1	1
If applicable, explain how missing data were handled in the analysis.	1	1	1	1	1	1
Summarize patient response rates and completeness of data collection.	1	1	1	1	1	1
Clarify what follow-up, if any, was expected and the percentage of patients for which incomplete data or follow-up was obtained.	1	1	1	1	1	1
Total score	10	10	10	10	10	10

**Table 3:** Study characteristics of the association between fruit and vegetable intake levels and the incidence of NAFLD were evaluated.

**References**	**Country**	**Research type**	**Total number of participants**	**Baseline age (years)**	**Gender (male/female)**	**Follow-up period (years)**	**Methods of disease diagnosis**	**Quality of study**
Chan et al. (27)	China	Cross-sectional study	797	36.2–60.3	332/465	/	Measurement of intrahepatic triglyceride content (IHTG) by 1H-MRS	Good
Liu et al. (28)	China	Cross-sectional study	1,639	18.55 ± 1.48	880/759	/	B-ultrasonic examination	Good
Tajima et al. (23)	Japan	Cross-sectional study	2,444	40–69	977/1,467	/	Abdominal ultrasonography	Good
Emamat et al. (24)	Iran	Case-control study	999	43.26 ± 14.03	430/569	/	Controlled attenuation parameter (CAP) score in Fibroscan exam	Good
Giraldi et al. (32)	Italy	Case-control study	815	51.37 ± 16.67	509/304	/	Presence of sonographic features of hepatic steatosis based on the presence of the bright liver pattern as recommended by the American Gastroenterology Association.	Good
Kim and Shin (25)	Korea	Cross-sectional study	52,280	40–79	15,588/36,692	4.2 years	NAFLD was diagnosed based on FLI Participants with FLI ≥60 were defined as having NAFLD.	Good
Noureddin et al. (29)	America	Case-control study	32,448	45–75	12,225/20,223	/	NAFLD cases among eligible participants were identified using Medicare claims	Good
Li et al. (33)	China	Cross-sectional study	26,891	≥18	12,727/14,164	/	Abdominal ultrasonography	Good
Tutunchi et al. (30)	Iran	Case-control study	210	30–60	90/120	/	Abdominal ultrasonography	Good
Du et al. (31)	China	Cross-sectional study	2,667	18–76	1,694/973	/	Abdominal ultrasonography	Good
Guo et al. (26)	UK	Case-control study	372,492	48.63–64.83	176,327/196,165	/	/	Good
465,15.5690pt**References**	**Sources of intake assessment**	**Adjustment factors**	**Relationship between vegetables or fruits and NAFLD OR (LL, UL)**	
			**Vegetables**	**Fruits**
Chan et al. (27)	FFQ	Age, sex, BMI, smoke, drink, central obesity, triglyceride >1.7 mmol/l, reduced HDL-cholesterol, hypertension, impaired fasting glucose or diabetes, the PNPLA3 genotypes (CC vs. CG vs. GG genotypes), and Energy intake	0.51 (0.3, 0.87)^*^	0.50 (0.3, 0.84)^*^
Liu et al. (28)	FFQ	Age, sex, BMI, economic income, smoking status, educational level, physical activity, family history of diabetes, stroke, and energy intake.	0.81 (0.66, 1.04)	0.84 (0.67, 1.07)
Tajima et al. (23)	BDHQ	Age, lifestyle factors, and BMI	0.83 (0.57, 1.21)	0.73 (0.5, 1.07)
Emamat et al. (24)	FFQ	Age, gender, BMI, energy intake, and physical activity	0.36 (0.22, 0.56)^*^	/
Giraldi et al. (32)	FFQ	Age, gender, total energy intake, diabetes status, smoking status, BMI, and physical activity.	1.81 (0.68, 4.78)	2.26 (0.97, 5.29)
Kim and Shin (25)	FFQ	Age, education level, smoking status, alcohol consumption, physical activity, energy intake, and red and processed meat intake, BMI	0.80 (0.69, 0.93)^*^	0.83 (0.72, 0.95)^*^
Noureddin et al. (29)	FFQ	BMI, alcohol intake, coffee intake, total soda intake, vigorous physical activity, and energy intake	0.99 (0.88, 1.1)	0.91 (0.81, 1.02)
Li et al. (33)	FFQ	Age, sex, smoking status, drinking status, education level, occupation, household income, physical activity, family history of disease (including cardiovascular disease, hypertension, hyperlipidemia, and diabetes), hypertension, hyperlipidemia, and diabetes, total energy intake, “fruits and sweet” dietary pattern score, “healthy dietary pattern score, and “animal foods” dietary pattern score, vegetable intake and fruit intake, BMI	0.81 (0.63, 1.05)	/
Tutunchi et al. (30)	Food diary	Sex, education, physical activity, BMI, and WC, the relationships and effect sizes for the residual effects of this variable	0.34 (0.16, 0.81)^*^	0.54 (0.19, 1.56)
Du et al. (31)	FFQ	Age, sex, educational attainment, BMI, WC, HC, BP, diabetes duration; family history, smoking, drinking, physical activity level, the consumption of bean products, salt, fish and sugary beverages, and biochemical index values (HbA1c, ALT, AST, and serum lipid levels).	0.67 (0.51, 0.88)^*^	1.15 (0.84, 1.59)
Guo et al. (26)	FFQ	Age, sex, race, education level, Townsend Deprivation Index (quartiles), drinking status, smoking status, exercise, BMI, and diabetes.	1.03 (0.93, 1.14)	0.89 (0.81, 0.98)^*^

^*^Indicates that the result is significant.

### 3.3 Results of the meta-analyses

#### 3.3.1 Vegetable intake

**Figure 2:**
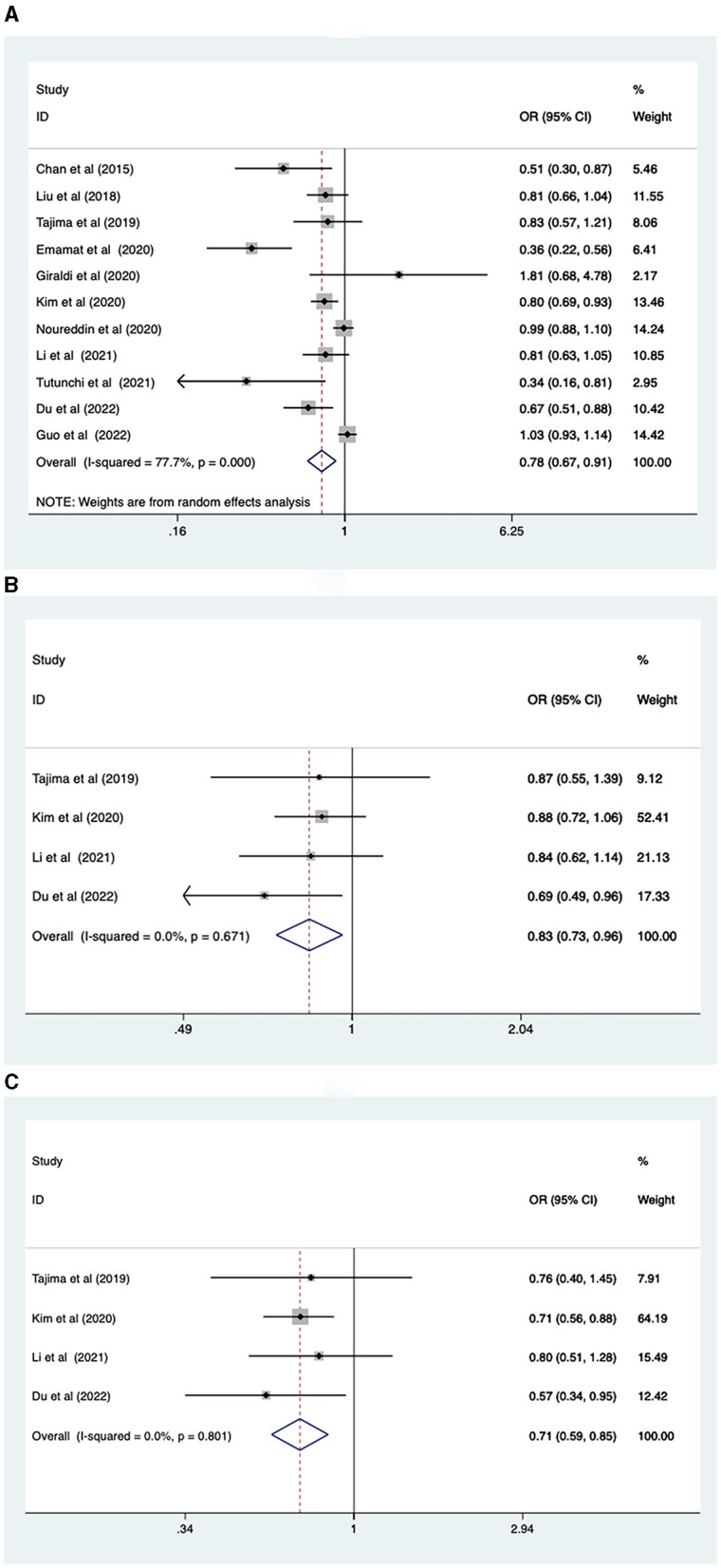
**(A)** Association between vegetable intake and risk of NAFLD; **(B)** NAFLD risk in men; **(C)** NAFLD risk in women.

Eleven studies involving 493,682 participants reported the association between vegetable intake and NAFLD risk. A random-effects model was used for data analysis (*I*^2^ = 77.7%, *p* < 0.001). The results found that higher vegetable intake was linked to a reduced risk of NAFLD (OR = 0.78, 95% Cl: 0.67–0.91, *p* = 0.001; Figure 2A).

Due to the greater heterogeneity in the analysis of total vegetable intake, subgroup analyses were conducted according to sex, study type, continent, and intake assessment questionnaire. The results revealed that increased levels of vegetable intake were associated with a reduced risk of NAFLD for both males (OR = 0.83, 95% CI: 0.73–0.96, *p* = 0.011), and females (OR = 0.71, 95% CI: 0.59–0.85, *p* < 0.001; Figures 2B, C). Regarding study types, higher levels of vegetable intake were connected with lower prevalence of NAFLD in cross-sectional studies (OR = 0.78, 95% CI: 0.70–0.86), *p* < 0.001. However, in case-control studies (OR = 0.79, 95% CI: 0.60–1.05, *p* = 0.107), no such association was observed (Supplementary Figure 1A). In terms of the geographic location of the study population, increased vegetable intake was inversely correlated with the risk of NAFLD in the Asian population (OR = 0.68, 95% CI: 0.58–0.82, *p* < 0.001). However, no correlation was found between vegetable intake and the risk of NAFLD in the European (OR = 1.10, 95%CI: 0.77–1.56, *p* = 0.6) and North American population (OR = 0.99, 95% CI: 0.89–1.11, *p* = 0.86; Supplementary Figure 1B). Regarding the intake assessment questionnaires, FFQ (OR = 0.80, 95% CI: 0.68–0.93, *p* = 0.005) and Food Diary (OR = 0.34, 95% CI: 0.15–0.77, *p* = 0.009) indicated that a higher vegetable intake was associated with decreased NAFLD risk. However, results from the BDHQ (OR = 0.83, 95% CI: 0.57–1.21, *p* = 0.332) showed the relationship between the two was not statistically significant (Supplementary Figure 1C).

When an adjustment factor was present in more than two studies, we conducted a single-factor heterogeneity analysis. Upon adjusting for age, gender, smoking and alcohol consumption status, physical activity, energy intake, BMI, economic income level, education level, and family history of diseases (hypertension, hyperlipidemia, cardiovascular disease, diabetes), we found that elevated levels of vegetable intake remained associated with a lower NAFLD risk (Supplementary Figures 2A–J). Yet, when we adjusted for hypertension, hyperlipidemia, diabetes, coffee intake, soft drinks, vegetable and fruit intake, and waist circumference, this association was not observed (Supplementary Figures 3A–G).

#### 3.3.2 Fruit intake

**Figure 3:**
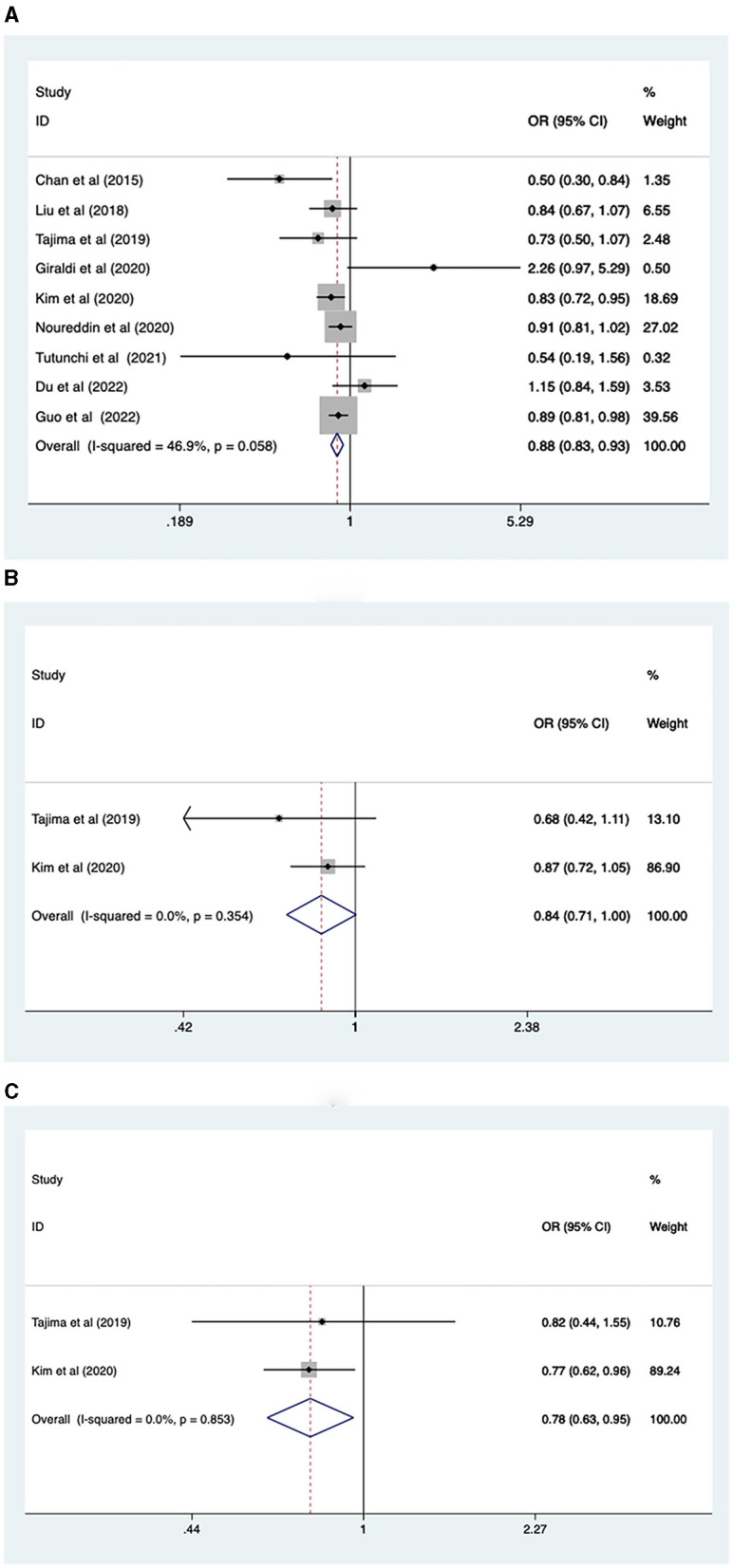
**(A)** Association between fruit intake and risk of NAFLD; **(B)** NAFLD risk in men; **(C)** NAFLD risk in women.

Nine studies, involving 465,792 participants, reported the association between fruit intake and NAFLD risk. A fixed-effects model was used (*I*^2^ = 46.9%, *p* = 0.058). The analysis revealed that higher levels of fruit intake were associated with a lower risk of NAFLD (OR = 0.88, 95% CI: 0.83–0.93, *p* < 0.001; Figure 3A).

Due to the heterogeneity of total fruit intake results, we performed a subgroup analysis based on sex, study type, continent, and intake assessment questionnaire. The fruit intake level was found to be associated with the prevalence of NAFLD in females (OR = 0.78, 95% CI: 0.63–0.95, *p* = 0.016), but not in males (OR = 0.84, 95% CI: 0.7–1.00; Figures 3B, C). Cross-sectional (OR = 0.84, 95% CI: 0.75, 0.93, *p* = 0.001) and case-control (OR = 0.90, 95% CI: 0.84, 0.97, *p* = 0.006) studies showed that higher levels of fruit intake were associated with lower NAFLD risk (Supplementary Figure 4A). In Asian (OR = 0.83, 95% CI: 0.75, 0.92, *p* = 0.001) and European (OR = 0.90, 95% CI: 0.82, 0.99, *p* = 0.03) populations, higher levels of fruit intake were associated with a lower risk of NAFLD, while in the North American population (OR = 0.91, 95% CI: 0.81, 1.02, *p* = 0.109), fruit intake was not associated with lower risk of NAFLD (Supplementary Figure 4B). FFQ (or = 0.89, 95% CI: 0.83, 0.94, *p* < 0.001) showed that a higher level of fruit intake was related to a lower level of NAFLD risk, while those of BDHQ (or = 0.73, 95% CI: 0.50, 1.07, *p* = 0.105) and Food Diary (or = 0.54, 95% CI: 0.19, 1.55, *p* = 0.251) showed that fruit intake had no significant impact on the risk of NAFLD (Supplementary Figure 4C).

Following adjustment for variables such as alcohol consumption, BMI, education level, energy intake, and physical activity, we observed that a high intake of fruits was still significantly associated with a reduced risk of NAFLD (Supplementary Figures 5A–E). However, further adjustments for factors such as age, gender, smoking status, coffee consumption, soft drink intake, history of diabetes, family history of diseases (hypertension, hyperlipidemia, cardiovascular disease, and diabetes), and waist circumference did not reveal any substantial correlation between fruit consumption and a lower risk of NAFLD (Supplementary Figures 6A–H).

### 3.4 Sensitivity analyses and publication bias

**Figure 4:**
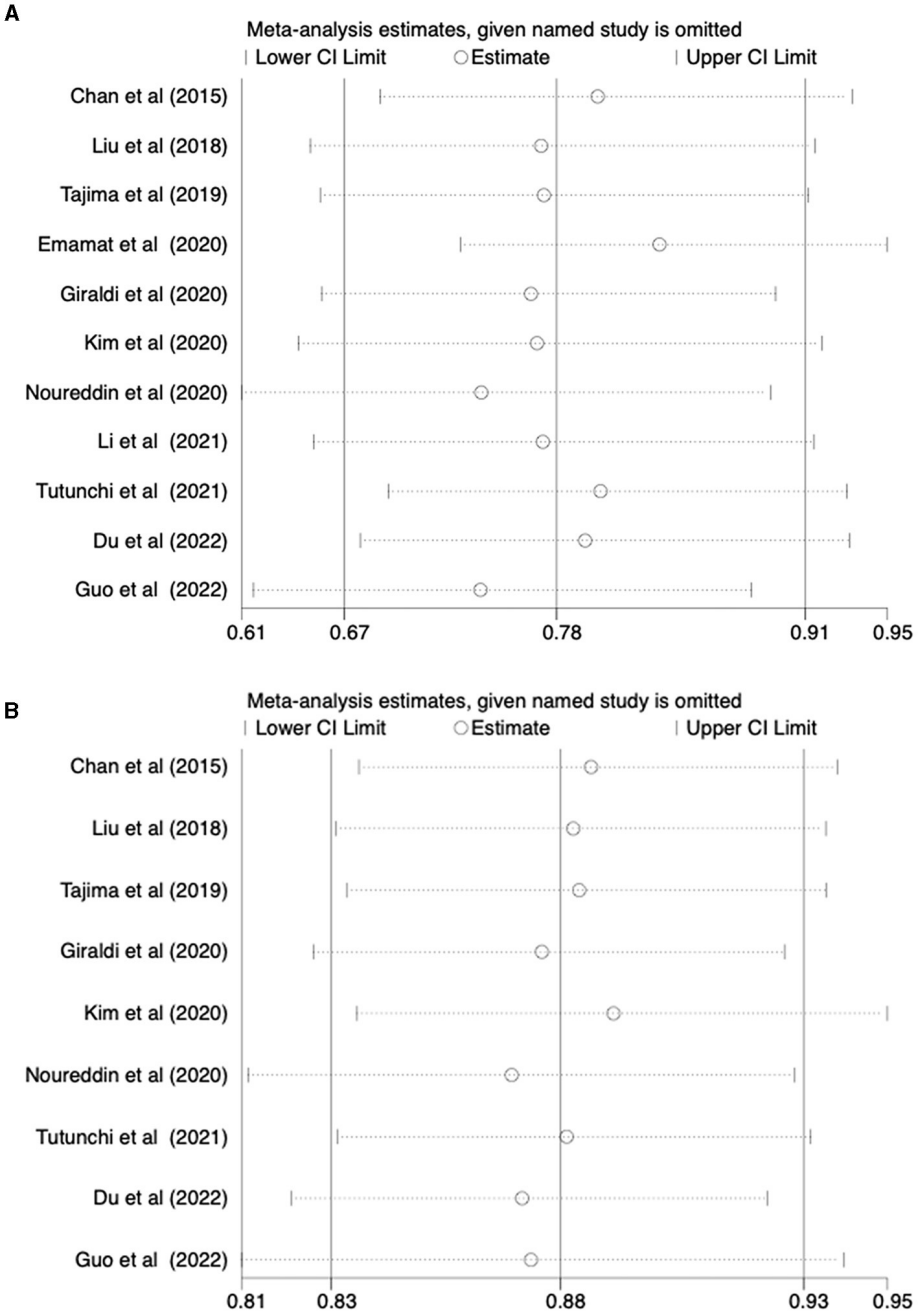
**(A)** Outcome sensitivity analysis of vegetable intake and risk of NAFLD; **(B)** Outcome sensitivity analysis of fruit intake and risk of NAFLD.

**Figure 5:**
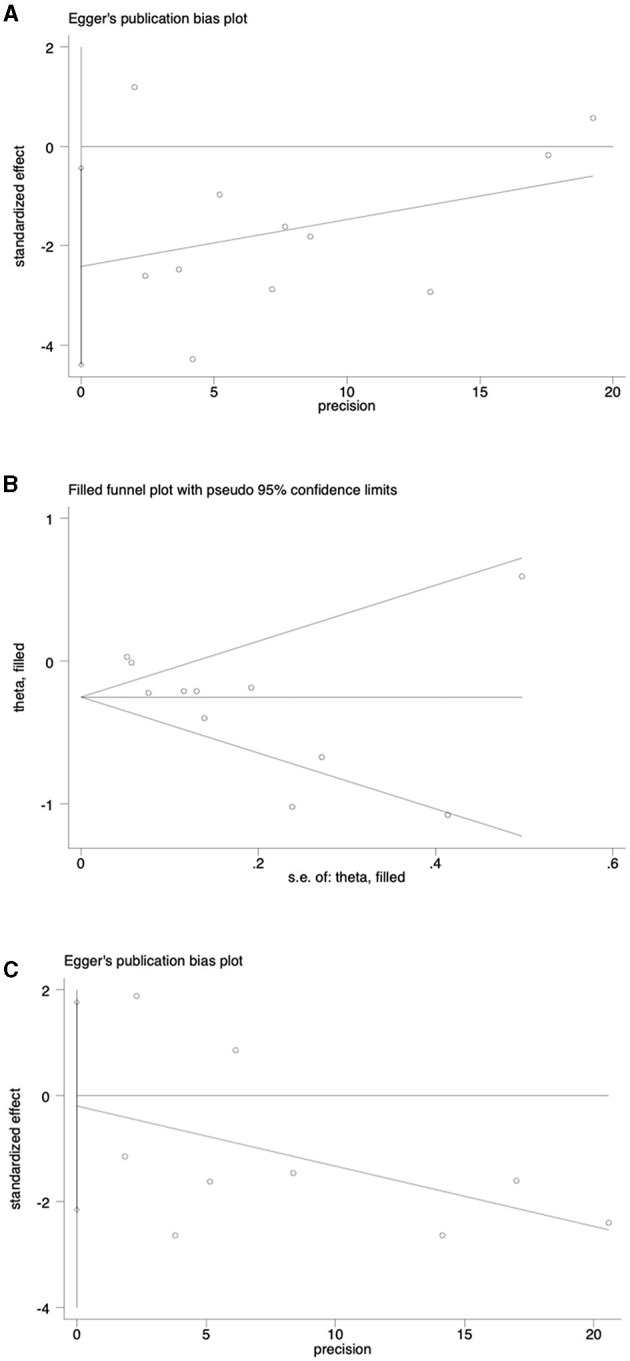
**(A)** Analysis of publication bias of outcomes associated with vegetable intake and risk of NAFLD; **(B)** Publication bias analysis was adjusted for the association between vegetable intake and NAFLD risk; **(C)** Analysis of publication bias for outcomes associated with fruit consumption and risk of NAFLD.

A sensitivity analysis was conducted to evaluate the stability of the results regarding the intake of vegetables or fruits and the risk of NAFLD. After excluding each article one by one, the results remained stable (Figure 4). To further examine publication bias, Egger's (*p* = 0.022) and Begg's (*p* = 0.161) tests were conducted for the relationship between vegetable intake and the risk of NAFLD. The Egger's test showed evidence of publication bias (Figure 5A). Hence, the Trim and Fill method was employed to adjust for the asymmetry in the funnel plot. However, the result indicated that no trimming was necessary and the data remained unchanged. When using Duval's Trim and Fill method, no new studies were added, suggesting that publication bias did not impact the study results (Figure 5B). Additionally, we analyzed publication bias using Egger's (*p* = 0.822) and Begg's (*p* = 0.754) tests for the relationship between fruit intake and the risk of NAFLD, finding no evidence of publication bias (Figure 5C).

## 4 Discussion

This represents the inaugural meta-analysis study investigating the connection between the intake of vegetables and fruits and the risk of NAFLD. A total of 11 studies involving 493,682 participants were included in the analysis, and the results suggest a negative correlation between the consumption of vegetables and fruits and the risk of NAFLD.

Dietary fiber, which is abundant in fruits and vegetables, is a type of short-chain fatty acids (SCFAs) produced by the fermentation of intestinal microorganisms in the gastrointestinal tract. These SCFAs, like propionic acid and butyric acid, have numerous benefits, including maintaining the integrity of the intestinal barrier, reducing the inflammatory reaction in the liver, regulating appetite, and maintaining glucose balance at the systemic level. This is all helpful in maintaining the energy balance of the liver, improving insulin sensitivity, and regulating liver lipid metabolism (34, 35). Furthermore, dietary fiber also enhances satiety, thus promoting calorie restriction (36). Additionally, fruits and vegetables are rich in antioxidants, including vitamin C, vitamin E, beta-carotene, polyphenols, which neutralize free radicals and mitigate the detrimental effects of oxidative stress on the liver (37–41). It should also be noted that NAFLD is frequently accompanied by an inflammatory response. Therefore, inhibiting inflammation is crucial to alleviating NAFLD symptoms. Fruits and vegetables are rich in polyphenols and flavonoids, which possess anti-inflammatory properties and can reduce the severity of inflammation in the liver (41, 42).

It is observed that in some of the included studies, the relationship between intake levels of vegetables (23, 26, 28, 29, 32) and fruits (23, 28, 30–33) and the prevalence of NAFLD were not consistent with the conclusion in this meta-analysis. In particular, the proportion of related studies on fruit intake with inconsistent conclusions with this meta-analysis is relatively high. This discrepancy may be attributed to variations in disease diagnosis methods, study populations, adjustment factors, and dietary assessment methods.

The diagnosis of NAFLD differed across the included studies, and differences in the definition of fatty liver and the degree of medical diagnosis may have led to discrepancies in conclusions. Chan's study (27) measured intrahepatic triglyceride content (IHTG) using 1H-MRS within 8 weeks after the baseline visit of included participants, and the result showed that higher levels of vegetable and fruit intake were associated with a lower prevalence of NAFLD. Liu et al. (28) used B-ultrasound as the diagnostic basis, Giraldi et al. (32) used bright liver pattern and ultrasound features of liver steatosis as the diagnostic basis, and Noureddin et al. (29) identified eligible NAFLD patients through Medicare claims. Their results showed that vegetable and fruit intake levels were not associated with the prevalence of NAFLD. It is worth discussing that in the study with a large sample size conducted in South Korea by Kim and Shin (25), the incidence risk of NAFLD in the female population was related to vegetable and fruit intake, while in the male population, only fruit intake was found to be related. In the overall population, the incidence risk of NAFLD was associated with the intake of both vegetables and fruits. However, this conclusion may have significant bias because the diagnosis of NAFLD is based on the Fatty Liver Index (FLI), using a cutoff value of 60. However, in the study by Kim et al., 46% of the participants were of normal weight or underweight, and the accuracy of FLI in diagnosing NAFLD in lean NAFLD patients was low. This is because patients with low body mass index and NAFLD would not be able to reach the FLI limit for diagnosing NAFLD in the absence of increased GGT or triglycerides (included in the FLI calculation) (43). Furthermore, the accuracy of the critical value of 60 for diagnosing NAFLD is low in Asian populations, including in South Korea, because after the study by Kim et al., the ideal critical value for South Koreans was described to be equal to 29 (44–46).

Furthermore, results may vary between populations. Asians may have a stronger preference for leafy vegetables such as spinach and cabbage, while Europeans and Americans tend to consume vegetables like corn, squash, potatoes, onions, and broccoli, which contain higher levels of starch. This dietary difference could explain the diverse findings regarding the correlation between vegetable and fruit intake and NAFLD among participants in Asia, Europe, and America. Notably, there is limited literature from Europe and the Americas, necessitating cautious interpretation of these results and future confirmation through additional relevant studies. Additionally, we observed different results between males and females (23, 25, 31, 33), which may be attributed to notable differences in their dietary patterns (33). Studies have shown that females tend to increase their intake of fruits and vegetables more than males (47, 48), and there are sex-specific disparities in fatty acid oxidation and regulation of liver *de novo* lipogenesis (DNL), with males inhibiting DNL less rapidly than females, leading to a shift in cellular metabolism from fatty acid oxidation to esterification (49). Since gender-specific studies are relatively scarce, further clinical data are required to validate these conclusions.

Results may vary when adjusting factors in a study. After adjusting for social and economic status and other factors in Li et al.'s study (33), green leafy vegetables (GLV) were negatively correlated with NAFLD. However, further adjustment for BMI eliminated this negative correlation. We found that adjusting for BMI did not significantly alter the results in multiple studies, including Noureddin et al.'s study (29). Although GLV intake is negatively correlated with NAFLD in normal/overweight individuals, obesity-related metabolic complications such as hyperlipidemia and insulin resistance may significantly increase liver lipids, resulting in decreased insulin sensitivity. These complications cannot be regulated by lipid metabolism and GLV intake (50). Additionally, some reports show that obese individuals significantly underestimate their dietary intake in self-recording or interview evaluation (51), which may explain why some studies did not observe the relationship between GLV intake and NAFLD after adjusting for BMI. Liu et al. (28) investigated the relationship between dietary patterns and NAFLD in Chinese adolescents, finding no association between fruit and vegetable intake and the occurrence of NAFLD. Teenagers usually have excellent physiological functions and efficiently absorb nutrients from food. Meanwhile, teenagers are more prone to unhealthy eating patterns that could affect their BMI and contribute to differences in conclusions. Therefore, additional studies are required to confirm the reliability of this conclusion.

In studies examining the intake levels of total vegetables and total fruits (25) and their association with the prevalence of NAFLD, results show a negative correlation. However, research by Liu et al. (28) and Giraldi et al. (32) indicates no relationship between vegetable and fruit intake levels and the prevalence of NAFLD. Guo et al.'s study (26) found significant results for fruit intake but not for vegetable intake. These studies included vegetables and fruits within dietary patterns, which are typically composed of independent or interactive foods and complex nutrient combinations that affect human metabolism. Therefore, it is challenging to exclude the synergistic effects of nutritional foods on NAFLD. Furthermore, not all types of vegetables and fruits are associated with a reduced risk of chronic diseases, as they contain different components and bioactive phytochemicals (52). While vegetables are generally considered low-carbohydrate foods and those with high dietary fiber levels may reduce the risk of NAFLD (33), excessive intake of starchy vegetables might increase blood glucose and insulin resistance, which is detrimental to NAFLD patients (53). Similarly, fruits contain natural fructose, and high fructose intake increases hepatic *de novo* lipogenesis (DNL), reduces fatty acid β-oxidation (FAO), and leads to fatty acid deposition (54). Excessive fructose intake can also promote the development of NAFLD. Additionally, the type of fructose—natural fructose from fruits vs. industrial fructose—might lead to different research conclusions. In Tajima et al.'s study (23), fruit intake was negatively correlated with the fatty liver index in the elderly, whose dietary fructose mainly came from fruits (55). Despite inquiries about fruit intake in studies, younger individuals might mistakenly count industrial fructose and fruit juice consumption as fruit intake. This could mean that, among the younger population, the harmful effects of industrial fructose and soft drinks on NAFLD might outweigh the protective effects of fruits on NAFLD (55). These factors may explain the inconsistencies between the conclusions regarding fruit intake in the included studies and the results of this meta-analysis.

In addition, there are some factors that cannot be avoided in the included studies. For example, in Giraldi et al. (32), there may be large differences in samples and high variability, resulting in wide confidence intervals, and outcomes may be affected. In Li et al.'s study (33), fruit intake was recorded for both NAFLD patients and the control group. However, the results were not significant, possibly due to differences in the study population, adjustment factors, dietary assessment methods, or recall bias in participants' reporting of fruit intake, leading to the omission of ORs. This does not meet the inclusion criteria for studies on fruit intake and NAFLD prevalence in our research and consequently contributes to a degree of selection bias in this study. Furthermore, the assessment questionnaire itself has self-reporting bias and subjectivity, and different assessment methods have different and limited contents, such as the lack of eating methods of vegetables and fruits, the choice of types of vegetables and fruits, the combination of food and the time of eating, which may also lead to different outcomes.

## 5 Strengths and limitations

Our study has various strengths. Firstly, it is the first meta-analysis to investigate the association between vegetable and fruit intake and incidence of NAFLD, utilizing large sample sizes. Secondly, in most of the studies included in the meta-analysis, the incidence of major NAFLD risk factors was controlled. Finally, we conducted subgroup analysis and stratification analysis of confounding factors to explore the sources of heterogeneity in the association between vegetable and fruit intake and NAFLD events.

Our study has a few limitations. Firstly, the included studies in our analysis encompassed case-control studies. As these studies employed food assessment questionnaires to estimate dietary intake, we cannot entirely rule out the possibility of measurement errors resulting from under- or over-reporting of food group intake due to participants' subjective judgments or memory biases. This could introduce recall and selection bias. Secondly, even though most studies adjusted for potential risk factors of NAFLD, residual confounding is always a concern in all observational studies. Finally, ORs were pooled from the highest and lowest intake levels, but intake levels were not always consistent across studies. Because of limited data, we were unable to include all studies in the dose-response analysis.

## 6 Conclusion

In conclusion, this systematic review and meta-analysis provide evidence that higher fruit and vegetable intake is linked to a lower risk of NAFLD. However, given that the relationship between vegetable intake and NAFLD incidence varies across different populations (age, sex, and ethnicity), types of vegetables, and fruits, more high-quality prospective studies are desired to further elucidate this connection. Additionally, there are studies suggesting that excessive fruit intake may actually promote the development of NAFLD.

## Data availability statement

The original contributions presented in the study are included in the article/Supplementary material, further inquiries can be directed to the corresponding authors.

## Author contributions

RW: Data curation, Formal analysis, Investigation, Methodology, Software, Visualization, Writing – original draft. RY: Data curation, Formal analysis, Methodology, Software, Writing – review & editing. JJ: Investigation, Project administration, Software, Writing – review & editing. FL: Data curation, Investigation, Software, Writing – review & editing. HZ: Investigation, Software, Visualization, Writing – review & editing. ZC: Conceptualization, Methodology, Resources, Supervision, Writing – review & editing. HW: Project administration, Supervision, Validation, Writing – review & editing. SY: Conceptualization, Methodology, Project administration, Supervision, Validation, Writing – review & editing. JL: Conceptualization, Funding acquisition, Methodology, Project administration, Resources, Supervision, Validation, Writing – review & editing.

## Supplementary material

Appendix 1 The complete retrieval formula.

Supplementary Figure 1 Subgroup of the association between vegetable intake and the risk of NAFLD. **(A)** Research type; **(B)** Nation state; **(C)** Assessment questionnaire.

Supplementary Figure 2 Stratified analysis of the association between vegetable intake and the risk of NAFLD (relevant). **(A)** age; **(B)** gender; **(C)** Smoking status; **(D)** Alcohol Consumption status; **(E)** physical exercise; **(F)** Energy intake; **(G)** BIM; **(H)** economic income; **(I)** educational level; **(J)** Family history of disease.

Supplementary Figure 3 Stratified analysis of the association between vegetable intake and the risk of NAFLD (irrelevant). **(A)** High blood pressure, high cholesterol; **(B)** diabetes; **(C)** Coffee intake; **(D)** Soft drink intake; **(E)** Vegetable intake; **(F)** Fruit intake; **(G)** waistline.

Supplementary Figure 4 Subgroup of the association between fruit consumption and the risk of NAFLD. **(A)** Study type heterogeneity; **(B)** National heterogeneity; **(C)** Heterogeneity of fruit intake was assessed.

Supplementary Figure 5 Stratified analyses of the association between fruit consumption and the risk of NAFLD (relevant). **(A)** Alcohol Consumption status; **(B)** BMI; **(C)** educational level; **(D)** energy intake; **(E)** physical exercise.

Supplementary Figure 6 Stratified analyses of the association between fruit consumption and the risk of NAFLD (irrelevant). **(A)** age; **(B)** gender; **(C)** smoking status; **(D)** Coffee intake; **(E)** Soft drink consumption; **(F)** diabetes; **(G)** Family history of disease; **(H)** waistline.
