# Supplementary material for: Fruit and vegetable intake and the risk of non-alcoholic fatty liver disease: a meta-analysis of observational studies
Source: Front Nutr. 2024 Jun 21;11:1398184. doi: 10.3389/fnut.2024.1398184 (PMC11224539; doi:10.3389/fnut.2024.1398184)
Supplement: Appendix 1 — The complete retrieval formula. [file Data_Sheet_1.ZIP › Supplementary materials/Appendix 1.docx]

**Appendix 1 Search strategy**

| 1. **PubMed retrieval style** | | | | | | |
| --- | --- | --- | --- | --- | --- | --- |
| Search number | Query | Sort By | Filters | Search Details | Results | Time |
| 7 | ((("Non-alcoholic Fatty Liver Disease"[Mesh]) OR (((((((((((((NAFLD[Title/Abstract]) OR (NASH[Title/Abstract])) OR (Nonalcoholic Fatty Liver*[Title/Abstract])) OR (Nonalcoholic Steatohepatiti*[Title/Abstract])) OR (non alcoholic hepat* steatosis[Title/Abstract])) OR (non alcoholic hepatosteatosis[Title/Abstract])) OR (non alcoholic liver steatosis[Title/Abstract])) OR (non alcoholic steatotic hepatopathy[Title/Abstract])) OR (non-alcoholic FLD[Title/Abstract])) OR (nonalcoholic FLD[Title/Abstract])) OR (nonalcoholic hepatic steatosis[Title/Abstract])) OR (nonalcoholic hepatosteatosis[Title/Abstract])) OR (nonalcoholic liver steatosis[Title/Abstract]))) AND ((((((((((((((((((((((((((((((((((((((((((((((((((((((((((((((((vegetable*[Title/Abstract]) OR (fruit*[Title/Abstract])) OR (diet*[Title/Abstract])) OR (nutrition[Title/Abstract])) OR (Potato*[Title/Abstract])) OR (manioc[Title/Abstract])) OR (cassava[Title/Abstract])) OR (taro[Title/Abstract])) OR (carrot*[Title/Abstract])) OR (beet*[Title/Abstract])) OR (parsnip*[Title/Abstract])) OR (turnip*[Title/Abstract])) OR (spinach[Title/Abstract])) OR (lettuce[Title/Abstract])) OR (cabbage*[Title/Abstract])) OR (broccoli[Title/Abstract])) OR (bok choy[Title/Abstract])) OR (watercress[Title/Abstract])) OR (allium[Title/Abstract])) OR (onion*[Title/Abstract])) OR (garlic[Title/Abstract])) OR (leek*[Title/Abstract])) OR (Apple*[Title/Abstract])) OR (banana*[Title/Abstract])) OR (berries[Title/Abstract])) OR (berry[Title/Abstract])) OR (grape*[Title/Abstract])) OR (citrus[Title/Abstract])) OR (orange*[Title/Abstract])) OR (grapefruit*[Title/Abstract])) OR (lemon*[Title/Abstract])) OR (lime*[Title/Abstract])) OR (apricot*[Title/Abstract])) OR (endive*[Title/Abstract])) OR (greens[Title/Abstract])) OR (romaine[Title/Abstract])) OR (brussels sprout*[Title/Abstract])) OR (cauliflower[Title/Abstract])) OR (kohlrabi[Title/Abstract])) OR (collard*[Title/Abstract])) OR (kale[Title/Abstract])) OR (rutabaga*[Title/Abstract])) OR (Umbelliferous[Title/Abstract])) OR (Celery[Title/Abstract])) OR (parsley[Title/Abstract])) OR (fennel[Title/Abstract])) OR (shallot*[Title/Abstract])) OR (chive[Title/Abstract])) OR (eggplant[Title/Abstract])) OR (tomato*[Title/Abstract])) OR (Gourd*[Title/Abstract])) OR (pumpkin*[Title/Abstract])) OR (squash[Title/Abstract])) OR (cucumber*[Title/Abstract])) OR (muskmelon*[Title/Abstract])) OR (watermelon*[Title/Abstract])) OR (melon*[Title/Abstract])) OR (Mustard[Title/Abstract])) OR (solanaceous[Title/Abstract])) OR (cucurbitaceous[Title/Abstract])) OR (sage[Title/Abstract])) OR (dill[Title/Abstract])) OR (mint[Title/Abstract])) OR (Cruciferous[Title/Abstract]))) NOT (mouse[Title/Abstract]) |  |  | (("Non-alcoholic Fatty Liver Disease"[MeSH Terms] OR ("NAFLD"[Title/Abstract] OR "NASH"[Title/Abstract] OR "nonalcoholic fatty liver*"[Title/Abstract] OR "nonalcoholic steatohepatiti*"[Title/Abstract] OR (("non"[All Fields] AND ("alcohol s"[All Fields] OR "alcoholate"[All Fields] OR "alcoholates"[All Fields] OR "alcoholic s"[All Fields] OR "alcoholics"[MeSH Terms] OR "alcoholics"[All Fields] OR "alcoholic"[All Fields] OR "alcoholism"[MeSH Terms] OR "alcoholism"[All Fields] OR "alcoholisms"[All Fields] OR "alcoholism s"[All Fields] OR "alcoholization"[All Fields] OR "alcohols"[MeSH Terms] OR "alcohols"[All Fields] OR "ethanol"[MeSH Terms] OR "ethanol"[All Fields] OR "alcohol"[All Fields]) AND "hepat*"[All Fields]) AND "steatosis"[Title/Abstract]) OR "non alcoholic hepatosteatosis"[Title/Abstract] OR "non alcoholic liver steatosis"[Title/Abstract] OR (("non"[All Fields] AND ("alcohol s"[All Fields] OR "alcoholate"[All Fields] OR "alcoholates"[All Fields] OR "alcoholic s"[All Fields] OR "alcoholics"[MeSH Terms] OR "alcoholics"[All Fields] OR "alcoholic"[All Fields] OR "alcoholism"[MeSH Terms] OR "alcoholism"[All Fields] OR "alcoholisms"[All Fields] OR "alcoholism s"[All Fields] OR "alcoholization"[All Fields] OR "alcohols"[MeSH Terms] OR "alcohols"[All Fields] OR "ethanol"[MeSH Terms] OR "ethanol"[All Fields] OR "alcohol"[All Fields]) AND "steatotic"[All Fields]) AND "hepatopathy"[Title/Abstract]) OR "non alcoholic fld"[Title/Abstract] OR "nonalcoholic fld"[Title/Abstract] OR "nonalcoholic hepatic steatosis"[Title/Abstract] OR "nonalcoholic hepatosteatosis"[Title/Abstract] OR "nonalcoholic liver steatosis"[Title/Abstract])) AND ("vegetable*"[Title/Abstract] OR "fruit*"[Title/Abstract] OR "diet*"[Title/Abstract] OR "nutrition"[Title/Abstract] OR "potato*"[Title/Abstract] OR "manioc"[Title/Abstract] OR "cassava"[Title/Abstract] OR "taro"[Title/Abstract] OR "carrot*"[Title/Abstract] OR "beet*"[Title/Abstract] OR "parsnip*"[Title/Abstract] OR "turnip*"[Title/Abstract] OR "spinach"[Title/Abstract] OR "lettuce"[Title/Abstract] OR "cabbage*"[Title/Abstract] OR "broccoli"[Title/Abstract] OR "bok choy"[Title/Abstract] OR "watercress"[Title/Abstract] OR "allium"[Title/Abstract] OR "onion*"[Title/Abstract] OR "garlic"[Title/Abstract] OR "leek*"[Title/Abstract] OR "apple*"[Title/Abstract] OR "banana*"[Title/Abstract] OR "berries"[Title/Abstract] OR "berry"[Title/Abstract] OR "grape*"[Title/Abstract] OR "citrus"[Title/Abstract] OR "orange*"[Title/Abstract] OR "grapefruit*"[Title/Abstract] OR "lemon*"[Title/Abstract] OR "lime*"[Title/Abstract] OR "apricot*"[Title/Abstract] OR "endive*"[Title/Abstract] OR "greens"[Title/Abstract] OR "romaine"[Title/Abstract] OR "brussels sprout*"[Title/Abstract] OR "cauliflower"[Title/Abstract] OR "kohlrabi"[Title/Abstract] OR "collard*"[Title/Abstract] OR "kale"[Title/Abstract] OR "rutabaga*"[Title/Abstract] OR "Umbelliferous"[Title/Abstract] OR "Celery"[Title/Abstract] OR "parsley"[Title/Abstract] OR "fennel"[Title/Abstract] OR "shallot*"[Title/Abstract] OR "chive"[Title/Abstract] OR "eggplant"[Title/Abstract] OR "tomato*"[Title/Abstract] OR "gourd*"[Title/Abstract] OR "pumpkin*"[Title/Abstract] OR "squash"[Title/Abstract] OR "cucumber*"[Title/Abstract] OR "muskmelon*"[Title/Abstract] OR "watermelon*"[Title/Abstract] OR "melon*"[Title/Abstract] OR "Mustard"[Title/Abstract] OR "solanaceous"[Title/Abstract] OR "cucurbitaceous"[Title/Abstract] OR "sage"[Title/Abstract] OR "dill"[Title/Abstract] OR "mint"[Title/Abstract] OR "Cruciferous"[Title/Abstract])) NOT "mouse"[Title/Abstract] | 9,230 | 5:38:36 |
| 6 | ((("Non-alcoholic Fatty Liver Disease"[Mesh]) OR (((((((((((((NAFLD[Title/Abstract]) OR (NASH[Title/Abstract])) OR (Nonalcoholic Fatty Liver*[Title/Abstract])) OR (Nonalcoholic Steatohepatiti*[Title/Abstract])) OR (non alcoholic hepat* steatosis[Title/Abstract])) OR (non alcoholic hepatosteatosis[Title/Abstract])) OR (non alcoholic liver steatosis[Title/Abstract])) OR (non alcoholic steatotic hepatopathy[Title/Abstract])) OR (non-alcoholic FLD[Title/Abstract])) OR (nonalcoholic FLD[Title/Abstract])) OR (nonalcoholic hepatic steatosis[Title/Abstract])) OR (nonalcoholic hepatosteatosis[Title/Abstract])) OR (nonalcoholic liver steatosis[Title/Abstract]))) AND ((((((((((((((((((((((((((((((((((((((((((((((((((((((((((((((((vegetable*[Title/Abstract]) OR (fruit*[Title/Abstract])) OR (diet*[Title/Abstract])) OR (nutrition[Title/Abstract])) OR (Potato*[Title/Abstract])) OR (manioc[Title/Abstract])) OR (cassava[Title/Abstract])) OR (taro[Title/Abstract])) OR (carrot*[Title/Abstract])) OR (beet*[Title/Abstract])) OR (parsnip*[Title/Abstract])) OR (turnip*[Title/Abstract])) OR (spinach[Title/Abstract])) OR (lettuce[Title/Abstract])) OR (cabbage*[Title/Abstract])) OR (broccoli[Title/Abstract])) OR (bok choy[Title/Abstract])) OR (watercress[Title/Abstract])) OR (allium[Title/Abstract])) OR (onion*[Title/Abstract])) OR (garlic[Title/Abstract])) OR (leek*[Title/Abstract])) OR (Apple*[Title/Abstract])) OR (banana*[Title/Abstract])) OR (berries[Title/Abstract])) OR (berry[Title/Abstract])) OR (grape*[Title/Abstract])) OR (citrus[Title/Abstract])) OR (orange*[Title/Abstract])) OR (grapefruit*[Title/Abstract])) OR (lemon*[Title/Abstract])) OR (lime*[Title/Abstract])) OR (apricot*[Title/Abstract])) OR (endive*[Title/Abstract])) OR (greens[Title/Abstract])) OR (romaine[Title/Abstract])) OR (brussels sprout*[Title/Abstract])) OR (cauliflower[Title/Abstract])) OR (kohlrabi[Title/Abstract])) OR (collard*[Title/Abstract])) OR (kale[Title/Abstract])) OR (rutabaga*[Title/Abstract])) OR (Umbelliferous[Title/Abstract])) OR (Celery[Title/Abstract])) OR (parsley[Title/Abstract])) OR (fennel[Title/Abstract])) OR (shallot*[Title/Abstract])) OR (chive[Title/Abstract])) OR (eggplant[Title/Abstract])) OR (tomato*[Title/Abstract])) OR (Gourd*[Title/Abstract])) OR (pumpkin*[Title/Abstract])) OR (squash[Title/Abstract])) OR (cucumber*[Title/Abstract])) OR (muskmelon*[Title/Abstract])) OR (watermelon*[Title/Abstract])) OR (melon*[Title/Abstract])) OR (Mustard[Title/Abstract])) OR (solanaceous[Title/Abstract])) OR (cucurbitaceous[Title/Abstract])) OR (sage[Title/Abstract])) OR (dill[Title/Abstract])) OR (mint[Title/Abstract])) OR (Cruciferous[Title/Abstract]))) NOT (meta[Title/Abstract]) |  |  | (("Non-alcoholic Fatty Liver Disease"[MeSH Terms] OR ("NAFLD"[Title/Abstract] OR "NASH"[Title/Abstract] OR "nonalcoholic fatty liver*"[Title/Abstract] OR "nonalcoholic steatohepatiti*"[Title/Abstract] OR (("non"[All Fields] AND ("alcohol s"[All Fields] OR "alcoholate"[All Fields] OR "alcoholates"[All Fields] OR "alcoholic s"[All Fields] OR "alcoholics"[MeSH Terms] OR "alcoholics"[All Fields] OR "alcoholic"[All Fields] OR "alcoholism"[MeSH Terms] OR "alcoholism"[All Fields] OR "alcoholisms"[All Fields] OR "alcoholism s"[All Fields] OR "alcoholization"[All Fields] OR "alcohols"[MeSH Terms] OR "alcohols"[All Fields] OR "ethanol"[MeSH Terms] OR "ethanol"[All Fields] OR "alcohol"[All Fields]) AND "hepat*"[All Fields]) AND "steatosis"[Title/Abstract]) OR "non alcoholic hepatosteatosis"[Title/Abstract] OR "non alcoholic liver steatosis"[Title/Abstract] OR (("non"[All Fields] AND ("alcohol s"[All Fields] OR "alcoholate"[All Fields] OR "alcoholates"[All Fields] OR "alcoholic s"[All Fields] OR "alcoholics"[MeSH Terms] OR "alcoholics"[All Fields] OR "alcoholic"[All Fields] OR "alcoholism"[MeSH Terms] OR "alcoholism"[All Fields] OR "alcoholisms"[All Fields] OR "alcoholism s"[All Fields] OR "alcoholization"[All Fields] OR "alcohols"[MeSH Terms] OR "alcohols"[All Fields] OR "ethanol"[MeSH Terms] OR "ethanol"[All Fields] OR "alcohol"[All Fields]) AND "steatotic"[All Fields]) AND "hepatopathy"[Title/Abstract]) OR "non alcoholic fld"[Title/Abstract] OR "nonalcoholic fld"[Title/Abstract] OR "nonalcoholic hepatic steatosis"[Title/Abstract] OR "nonalcoholic hepatosteatosis"[Title/Abstract] OR "nonalcoholic liver steatosis"[Title/Abstract])) AND ("vegetable*"[Title/Abstract] OR "fruit*"[Title/Abstract] OR "diet*"[Title/Abstract] OR "nutrition"[Title/Abstract] OR "potato*"[Title/Abstract] OR "manioc"[Title/Abstract] OR "cassava"[Title/Abstract] OR "taro"[Title/Abstract] OR "carrot*"[Title/Abstract] OR "beet*"[Title/Abstract] OR "parsnip*"[Title/Abstract] OR "turnip*"[Title/Abstract] OR "spinach"[Title/Abstract] OR "lettuce"[Title/Abstract] OR "cabbage*"[Title/Abstract] OR "broccoli"[Title/Abstract] OR "bok choy"[Title/Abstract] OR "watercress"[Title/Abstract] OR "allium"[Title/Abstract] OR "onion*"[Title/Abstract] OR "garlic"[Title/Abstract] OR "leek*"[Title/Abstract] OR "apple*"[Title/Abstract] OR "banana*"[Title/Abstract] OR "berries"[Title/Abstract] OR "berry"[Title/Abstract] OR "grape*"[Title/Abstract] OR "citrus"[Title/Abstract] OR "orange*"[Title/Abstract] OR "grapefruit*"[Title/Abstract] OR "lemon*"[Title/Abstract] OR "lime*"[Title/Abstract] OR "apricot*"[Title/Abstract] OR "endive*"[Title/Abstract] OR "greens"[Title/Abstract] OR "romaine"[Title/Abstract] OR "brussels sprout*"[Title/Abstract] OR "cauliflower"[Title/Abstract] OR "kohlrabi"[Title/Abstract] OR "collard*"[Title/Abstract] OR "kale"[Title/Abstract] OR "rutabaga*"[Title/Abstract] OR "Umbelliferous"[Title/Abstract] OR "Celery"[Title/Abstract] OR "parsley"[Title/Abstract] OR "fennel"[Title/Abstract] OR "shallot*"[Title/Abstract] OR "chive"[Title/Abstract] OR "eggplant"[Title/Abstract] OR "tomato*"[Title/Abstract] OR "gourd*"[Title/Abstract] OR "pumpkin*"[Title/Abstract] OR "squash"[Title/Abstract] OR "cucumber*"[Title/Abstract] OR "muskmelon*"[Title/Abstract] OR "watermelon*"[Title/Abstract] OR "melon*"[Title/Abstract] OR "Mustard"[Title/Abstract] OR "solanaceous"[Title/Abstract] OR "cucurbitaceous"[Title/Abstract] OR "sage"[Title/Abstract] OR "dill"[Title/Abstract] OR "mint"[Title/Abstract] OR "Cruciferous"[Title/Abstract])) NOT "meta"[Title/Abstract] | 10,968 | 4:55:45 |
| 5 | (("Non-alcoholic Fatty Liver Disease"[Mesh]) OR (((((((((((((NAFLD[Title/Abstract]) OR (NASH[Title/Abstract])) OR (Nonalcoholic Fatty Liver*[Title/Abstract])) OR (Nonalcoholic Steatohepatiti*[Title/Abstract])) OR (non alcoholic hepat* steatosis[Title/Abstract])) OR (non alcoholic hepatosteatosis[Title/Abstract])) OR (non alcoholic liver steatosis[Title/Abstract])) OR (non alcoholic steatotic hepatopathy[Title/Abstract])) OR (non-alcoholic FLD[Title/Abstract])) OR (nonalcoholic FLD[Title/Abstract])) OR (nonalcoholic hepatic steatosis[Title/Abstract])) OR (nonalcoholic hepatosteatosis[Title/Abstract])) OR (nonalcoholic liver steatosis[Title/Abstract]))) AND ((((((((((((((((((((((((((((((((((((((((((((((((((((((((((((((((vegetable*[Title/Abstract]) OR (fruit*[Title/Abstract])) OR (diet*[Title/Abstract])) OR (nutrition[Title/Abstract])) OR (Potato*[Title/Abstract])) OR (manioc[Title/Abstract])) OR (cassava[Title/Abstract])) OR (taro[Title/Abstract])) OR (carrot*[Title/Abstract])) OR (beet*[Title/Abstract])) OR (parsnip*[Title/Abstract])) OR (turnip*[Title/Abstract])) OR (spinach[Title/Abstract])) OR (lettuce[Title/Abstract])) OR (cabbage*[Title/Abstract])) OR (broccoli[Title/Abstract])) OR (bok choy[Title/Abstract])) OR (watercress[Title/Abstract])) OR (allium[Title/Abstract])) OR (onion*[Title/Abstract])) OR (garlic[Title/Abstract])) OR (leek*[Title/Abstract])) OR (Apple*[Title/Abstract])) OR (banana*[Title/Abstract])) OR (berries[Title/Abstract])) OR (berry[Title/Abstract])) OR (grape*[Title/Abstract])) OR (citrus[Title/Abstract])) OR (orange*[Title/Abstract])) OR (grapefruit*[Title/Abstract])) OR (lemon*[Title/Abstract])) OR (lime*[Title/Abstract])) OR (apricot*[Title/Abstract])) OR (endive*[Title/Abstract])) OR (greens[Title/Abstract])) OR (romaine[Title/Abstract])) OR (brussels sprout*[Title/Abstract])) OR (cauliflower[Title/Abstract])) OR (kohlrabi[Title/Abstract])) OR (collard*[Title/Abstract])) OR (kale[Title/Abstract])) OR (rutabaga*[Title/Abstract])) OR (Umbelliferous[Title/Abstract])) OR (Celery[Title/Abstract])) OR (parsley[Title/Abstract])) OR (fennel[Title/Abstract])) OR (shallot*[Title/Abstract])) OR (chive[Title/Abstract])) OR (eggplant[Title/Abstract])) OR (tomato*[Title/Abstract])) OR (Gourd*[Title/Abstract])) OR (pumpkin*[Title/Abstract])) OR (squash[Title/Abstract])) OR (cucumber*[Title/Abstract])) OR (muskmelon*[Title/Abstract])) OR (watermelon*[Title/Abstract])) OR (melon*[Title/Abstract])) OR (Mustard[Title/Abstract])) OR (solanaceous[Title/Abstract])) OR (cucurbitaceous[Title/Abstract])) OR (sage[Title/Abstract])) OR (dill[Title/Abstract])) OR (mint[Title/Abstract])) OR (Cruciferous[Title/Abstract])) |  |  | ("Non-alcoholic Fatty Liver Disease"[MeSH Terms] OR ("NAFLD"[Title/Abstract] OR "NASH"[Title/Abstract] OR "nonalcoholic fatty liver*"[Title/Abstract] OR "nonalcoholic steatohepatiti*"[Title/Abstract] OR (("non"[All Fields] AND ("alcohol s"[All Fields] OR "alcoholate"[All Fields] OR "alcoholates"[All Fields] OR "alcoholic s"[All Fields] OR "alcoholics"[MeSH Terms] OR "alcoholics"[All Fields] OR "alcoholic"[All Fields] OR "alcoholism"[MeSH Terms] OR "alcoholism"[All Fields] OR "alcoholisms"[All Fields] OR "alcoholism s"[All Fields] OR "alcoholization"[All Fields] OR "alcohols"[MeSH Terms] OR "alcohols"[All Fields] OR "ethanol"[MeSH Terms] OR "ethanol"[All Fields] OR "alcohol"[All Fields]) AND "hepat*"[All Fields]) AND "steatosis"[Title/Abstract]) OR "non alcoholic hepatosteatosis"[Title/Abstract] OR "non alcoholic liver steatosis"[Title/Abstract] OR (("non"[All Fields] AND ("alcohol s"[All Fields] OR "alcoholate"[All Fields] OR "alcoholates"[All Fields] OR "alcoholic s"[All Fields] OR "alcoholics"[MeSH Terms] OR "alcoholics"[All Fields] OR "alcoholic"[All Fields] OR "alcoholism"[MeSH Terms] OR "alcoholism"[All Fields] OR "alcoholisms"[All Fields] OR "alcoholism s"[All Fields] OR "alcoholization"[All Fields] OR "alcohols"[MeSH Terms] OR "alcohols"[All Fields] OR "ethanol"[MeSH Terms] OR "ethanol"[All Fields] OR "alcohol"[All Fields]) AND "steatotic"[All Fields]) AND "hepatopathy"[Title/Abstract]) OR "non alcoholic fld"[Title/Abstract] OR "nonalcoholic fld"[Title/Abstract] OR "nonalcoholic hepatic steatosis"[Title/Abstract] OR "nonalcoholic hepatosteatosis"[Title/Abstract] OR "nonalcoholic liver steatosis"[Title/Abstract])) AND ("vegetable*"[Title/Abstract] OR "fruit*"[Title/Abstract] OR "diet*"[Title/Abstract] OR "nutrition"[Title/Abstract] OR "potato*"[Title/Abstract] OR "manioc"[Title/Abstract] OR "cassava"[Title/Abstract] OR "taro"[Title/Abstract] OR "carrot*"[Title/Abstract] OR "beet*"[Title/Abstract] OR "parsnip*"[Title/Abstract] OR "turnip*"[Title/Abstract] OR "spinach"[Title/Abstract] OR "lettuce"[Title/Abstract] OR "cabbage*"[Title/Abstract] OR "broccoli"[Title/Abstract] OR "bok choy"[Title/Abstract] OR "watercress"[Title/Abstract] OR "allium"[Title/Abstract] OR "onion*"[Title/Abstract] OR "garlic"[Title/Abstract] OR "leek*"[Title/Abstract] OR "apple*"[Title/Abstract] OR "banana*"[Title/Abstract] OR "berries"[Title/Abstract] OR "berry"[Title/Abstract] OR "grape*"[Title/Abstract] OR "citrus"[Title/Abstract] OR "orange*"[Title/Abstract] OR "grapefruit*"[Title/Abstract] OR "lemon*"[Title/Abstract] OR "lime*"[Title/Abstract] OR "apricot*"[Title/Abstract] OR "endive*"[Title/Abstract] OR "greens"[Title/Abstract] OR "romaine"[Title/Abstract] OR "brussels sprout*"[Title/Abstract] OR "cauliflower"[Title/Abstract] OR "kohlrabi"[Title/Abstract] OR "collard*"[Title/Abstract] OR "kale"[Title/Abstract] OR "rutabaga*"[Title/Abstract] OR "Umbelliferous"[Title/Abstract] OR "Celery"[Title/Abstract] OR "parsley"[Title/Abstract] OR "fennel"[Title/Abstract] OR "shallot*"[Title/Abstract] OR "chive"[Title/Abstract] OR "eggplant"[Title/Abstract] OR "tomato*"[Title/Abstract] OR "gourd*"[Title/Abstract] OR "pumpkin*"[Title/Abstract] OR "squash"[Title/Abstract] OR "cucumber*"[Title/Abstract] OR "muskmelon*"[Title/Abstract] OR "watermelon*"[Title/Abstract] OR "melon*"[Title/Abstract] OR "Mustard"[Title/Abstract] OR "solanaceous"[Title/Abstract] OR "cucurbitaceous"[Title/Abstract] OR "sage"[Title/Abstract] OR "dill"[Title/Abstract] OR "mint"[Title/Abstract] OR "Cruciferous"[Title/Abstract]) | 11,070 | 4:45:07 |
| 4 | (((((((((((((((((((((((((((((((((((((((((((((((((((((((((((((((vegetable*[Title/Abstract]) OR (fruit*[Title/Abstract])) OR (diet*[Title/Abstract])) OR (nutrition[Title/Abstract])) OR (Potato*[Title/Abstract])) OR (manioc[Title/Abstract])) OR (cassava[Title/Abstract])) OR (taro[Title/Abstract])) OR (carrot*[Title/Abstract])) OR (beet*[Title/Abstract])) OR (parsnip*[Title/Abstract])) OR (turnip*[Title/Abstract])) OR (spinach[Title/Abstract])) OR (lettuce[Title/Abstract])) OR (cabbage*[Title/Abstract])) OR (broccoli[Title/Abstract])) OR (bok choy[Title/Abstract])) OR (watercress[Title/Abstract])) OR (allium[Title/Abstract])) OR (onion*[Title/Abstract])) OR (garlic[Title/Abstract])) OR (leek*[Title/Abstract])) OR (Apple*[Title/Abstract])) OR (banana*[Title/Abstract])) OR (berries[Title/Abstract])) OR (berry[Title/Abstract])) OR (grape*[Title/Abstract])) OR (citrus[Title/Abstract])) OR (orange*[Title/Abstract])) OR (grapefruit*[Title/Abstract])) OR (lemon*[Title/Abstract])) OR (lime*[Title/Abstract])) OR (apricot*[Title/Abstract])) OR (endive*[Title/Abstract])) OR (greens[Title/Abstract])) OR (romaine[Title/Abstract])) OR (brussels sprout*[Title/Abstract])) OR (cauliflower[Title/Abstract])) OR (kohlrabi[Title/Abstract])) OR (collard*[Title/Abstract])) OR (kale[Title/Abstract])) OR (rutabaga*[Title/Abstract])) OR (Umbelliferous[Title/Abstract])) OR (Celery[Title/Abstract])) OR (parsley[Title/Abstract])) OR (fennel[Title/Abstract])) OR (shallot*[Title/Abstract])) OR (chive[Title/Abstract])) OR (eggplant[Title/Abstract])) OR (tomato*[Title/Abstract])) OR (Gourd*[Title/Abstract])) OR (pumpkin*[Title/Abstract])) OR (squash[Title/Abstract])) OR (cucumber*[Title/Abstract])) OR (muskmelon*[Title/Abstract])) OR (watermelon*[Title/Abstract])) OR (melon*[Title/Abstract])) OR (Mustard[Title/Abstract])) OR (solanaceous[Title/Abstract])) OR (cucurbitaceous[Title/Abstract])) OR (sage[Title/Abstract])) OR (dill[Title/Abstract])) OR (mint[Title/Abstract])) OR (Cruciferous[Title/Abstract]) |  |  | "vegetable*"[Title/Abstract] OR "fruit*"[Title/Abstract] OR "diet*"[Title/Abstract] OR "nutrition"[Title/Abstract] OR "potato*"[Title/Abstract] OR "manioc"[Title/Abstract] OR "cassava"[Title/Abstract] OR "taro"[Title/Abstract] OR "carrot*"[Title/Abstract] OR "beet*"[Title/Abstract] OR "parsnip*"[Title/Abstract] OR "turnip*"[Title/Abstract] OR "spinach"[Title/Abstract] OR "lettuce"[Title/Abstract] OR "cabbage*"[Title/Abstract] OR "broccoli"[Title/Abstract] OR "bok choy"[Title/Abstract] OR "watercress"[Title/Abstract] OR "allium"[Title/Abstract] OR "onion*"[Title/Abstract] OR "garlic"[Title/Abstract] OR "leek*"[Title/Abstract] OR "apple*"[Title/Abstract] OR "banana*"[Title/Abstract] OR "berries"[Title/Abstract] OR "berry"[Title/Abstract] OR "grape*"[Title/Abstract] OR "citrus"[Title/Abstract] OR "orange*"[Title/Abstract] OR "grapefruit*"[Title/Abstract] OR "lemon*"[Title/Abstract] OR "lime*"[Title/Abstract] OR "apricot*"[Title/Abstract] OR "endive*"[Title/Abstract] OR "greens"[Title/Abstract] OR "romaine"[Title/Abstract] OR "brussels sprout*"[Title/Abstract] OR "cauliflower"[Title/Abstract] OR "kohlrabi"[Title/Abstract] OR "collard*"[Title/Abstract] OR "kale"[Title/Abstract] OR "rutabaga*"[Title/Abstract] OR "Umbelliferous"[Title/Abstract] OR "Celery"[Title/Abstract] OR "parsley"[Title/Abstract] OR "fennel"[Title/Abstract] OR "shallot*"[Title/Abstract] OR "chive"[Title/Abstract] OR "eggplant"[Title/Abstract] OR "tomato*"[Title/Abstract] OR "gourd*"[Title/Abstract] OR "pumpkin*"[Title/Abstract] OR "squash"[Title/Abstract] OR "cucumber*"[Title/Abstract] OR "muskmelon*"[Title/Abstract] OR "watermelon*"[Title/Abstract] OR "melon*"[Title/Abstract] OR "Mustard"[Title/Abstract] OR "solanaceous"[Title/Abstract] OR "cucurbitaceous"[Title/Abstract] OR "sage"[Title/Abstract] OR "dill"[Title/Abstract] OR "mint"[Title/Abstract] OR "Cruciferous"[Title/Abstract] | 1,188,983 | 3:08:20 |
| 3 | ("Non-alcoholic Fatty Liver Disease"[Mesh]) OR (((((((((((((NAFLD[Title/Abstract]) OR (NASH[Title/Abstract])) OR (Nonalcoholic Fatty Liver*[Title/Abstract])) OR (Nonalcoholic Steatohepatiti*[Title/Abstract])) OR (non alcoholic hepat* steatosis[Title/Abstract])) OR (non alcoholic hepatosteatosis[Title/Abstract])) OR (non alcoholic liver steatosis[Title/Abstract])) OR (non alcoholic steatotic hepatopathy[Title/Abstract])) OR (non-alcoholic FLD[Title/Abstract])) OR (nonalcoholic FLD[Title/Abstract])) OR (nonalcoholic hepatic steatosis[Title/Abstract])) OR (nonalcoholic hepatosteatosis[Title/Abstract])) OR (nonalcoholic liver steatosis[Title/Abstract])) |  |  | "Non-alcoholic Fatty Liver Disease"[MeSH Terms] OR ("NAFLD"[Title/Abstract] OR "NASH"[Title/Abstract] OR "nonalcoholic fatty liver*"[Title/Abstract] OR "nonalcoholic steatohepatiti*"[Title/Abstract] OR (("non"[All Fields] AND ("alcohol s"[All Fields] OR "alcoholate"[All Fields] OR "alcoholates"[All Fields] OR "alcoholic s"[All Fields] OR "alcoholics"[MeSH Terms] OR "alcoholics"[All Fields] OR "alcoholic"[All Fields] OR "alcoholism"[MeSH Terms] OR "alcoholism"[All Fields] OR "alcoholisms"[All Fields] OR "alcoholism s"[All Fields] OR "alcoholization"[All Fields] OR "alcohols"[MeSH Terms] OR "alcohols"[All Fields] OR "ethanol"[MeSH Terms] OR "ethanol"[All Fields] OR "alcohol"[All Fields]) AND "hepat*"[All Fields]) AND "steatosis"[Title/Abstract]) OR "non alcoholic hepatosteatosis"[Title/Abstract] OR "non alcoholic liver steatosis"[Title/Abstract] OR (("non"[All Fields] AND ("alcohol s"[All Fields] OR "alcoholate"[All Fields] OR "alcoholates"[All Fields] OR "alcoholic s"[All Fields] OR "alcoholics"[MeSH Terms] OR "alcoholics"[All Fields] OR "alcoholic"[All Fields] OR "alcoholism"[MeSH Terms] OR "alcoholism"[All Fields] OR "alcoholisms"[All Fields] OR "alcoholism s"[All Fields] OR "alcoholization"[All Fields] OR "alcohols"[MeSH Terms] OR "alcohols"[All Fields] OR "ethanol"[MeSH Terms] OR "ethanol"[All Fields] OR "alcohol"[All Fields]) AND "steatotic"[All Fields]) AND "hepatopathy"[Title/Abstract]) OR "non alcoholic fld"[Title/Abstract] OR "nonalcoholic fld"[Title/Abstract] OR "nonalcoholic hepatic steatosis"[Title/Abstract] OR "nonalcoholic hepatosteatosis"[Title/Abstract] OR "nonalcoholic liver steatosis"[Title/Abstract]) | 39,359 | 0:00:51 |
| 2 | ((((((((((((NAFLD[Title/Abstract]) OR (NASH[Title/Abstract])) OR (Nonalcoholic Fatty Liver*[Title/Abstract])) OR (Nonalcoholic Steatohepatiti*[Title/Abstract])) OR (non alcoholic hepat* steatosis[Title/Abstract])) OR (non alcoholic hepatosteatosis[Title/Abstract])) OR (non alcoholic liver steatosis[Title/Abstract])) OR (non alcoholic steatotic hepatopathy[Title/Abstract])) OR (non-alcoholic FLD[Title/Abstract])) OR (nonalcoholic FLD[Title/Abstract])) OR (nonalcoholic hepatic steatosis[Title/Abstract])) OR (nonalcoholic hepatosteatosis[Title/Abstract])) OR (nonalcoholic liver steatosis[Title/Abstract]) |  |  | "NAFLD"[Title/Abstract] OR "NASH"[Title/Abstract] OR "nonalcoholic fatty liver*"[Title/Abstract] OR "nonalcoholic steatohepatiti*"[Title/Abstract] OR (("non"[All Fields] AND ("alcohol s"[All Fields] OR "alcoholate"[All Fields] OR "alcoholates"[All Fields] OR "alcoholic s"[All Fields] OR "alcoholics"[MeSH Terms] OR "alcoholics"[All Fields] OR "alcoholic"[All Fields] OR "alcoholism"[MeSH Terms] OR "alcoholism"[All Fields] OR "alcoholisms"[All Fields] OR "alcoholism s"[All Fields] OR "alcoholization"[All Fields] OR "alcohols"[MeSH Terms] OR "alcohols"[All Fields] OR "ethanol"[MeSH Terms] OR "ethanol"[All Fields] OR "alcohol"[All Fields]) AND "hepat*"[All Fields]) AND "steatosis"[Title/Abstract]) OR "non alcoholic hepatosteatosis"[Title/Abstract] OR "non alcoholic liver steatosis"[Title/Abstract] OR (("non"[All Fields] AND ("alcohol s"[All Fields] OR "alcoholate"[All Fields] OR "alcoholates"[All Fields] OR "alcoholic s"[All Fields] OR "alcoholics"[MeSH Terms] OR "alcoholics"[All Fields] OR "alcoholic"[All Fields] OR "alcoholism"[MeSH Terms] OR "alcoholism"[All Fields] OR "alcoholisms"[All Fields] OR "alcoholism s"[All Fields] OR "alcoholization"[All Fields] OR "alcohols"[MeSH Terms] OR "alcohols"[All Fields] OR "ethanol"[MeSH Terms] OR "ethanol"[All Fields] OR "alcohol"[All Fields]) AND "steatotic"[All Fields]) AND "hepatopathy"[Title/Abstract]) OR "non alcoholic fld"[Title/Abstract] OR "nonalcoholic fld"[Title/Abstract] OR "nonalcoholic hepatic steatosis"[Title/Abstract] OR "nonalcoholic hepatosteatosis"[Title/Abstract] OR "nonalcoholic liver steatosis"[Title/Abstract] | 36,628 | 23:46:19 |
| 1 | "Non-alcoholic Fatty Liver Disease"[Mesh] | Most Recent |  | "Non-alcoholic Fatty Liver Disease"[MeSH Terms] | 22,036 | 23:43:59 |

| 1. **The retrieval method of Embase** | | | |
| --- | --- | --- | --- |
| No. | Query | Results | Date |
| #7 | #5 NOT #6 | 781 | 2-Mar-23 |
| #6 | mouse:ab,ti | 907779 | 2-Mar-23 |
| #5 | #3 AND #4 | 833 | 2-Mar-23 |
| #4 | vegetable*:ab,ti OR fruit*:ab,ti OR potato*:ab,ti OR manioc:ab,ti OR cassava:ab,ti OR taro:ab,ti OR carrot*:ab,ti OR beet*:ab,ti OR parsnip*:ab,ti OR turnip*:ab,ti OR spinach:ab,ti OR lettuce:ab,ti OR cabbage*:ab,ti OR 'bok choy':ab,ti OR broccoli:ab,ti OR watercress:ab,ti OR allium:ab,ti OR onion*:ab,ti OR garlic:ab,ti OR leek*:ab,ti OR apple*:ab,ti OR banana*:ab,ti OR berries:ab,ti OR berry:ab,ti OR grape*:ab,ti OR citrus:ab,ti OR orange*:ab,ti OR grapefruit*:ab,ti OR lemon*:ab,ti OR lime*:ab,ti OR apricot*:ab,ti OR endive*:ab,ti OR greens:ab,ti OR romaine:ab,ti OR 'brussels sprout*':ab,ti OR cauliflower:ab,ti OR kohlrabi:ab,ti OR collard*:ab,ti OR kale:ab,ti OR rutabaga*:ab,ti OR umbelliferous:ab,ti OR celery:ab,ti OR parsley:ab,ti OR fennel:ab,ti OR shallot*:ab,ti OR chive:ab,ti OR eggplant:ab,ti OR tomato*:ab,ti OR gourd*:ab,ti OR pumpkin*:ab,ti OR squash:ab,ti OR cucumber*:ab,ti OR muskmelon*:ab,ti OR watermelon*:ab,ti OR melon*:ab,ti OR mustard:ab,ti OR solanaceous:ab,ti OR cucurbitaceous:ab,ti OR sage:ab,ti OR dill:ab,ti OR mint:ab,ti OR cruciferous:ab,ti | 475740 | 2-Mar-23 |
| #3 | #1 OR #2 | 76065 | 2-Mar-23 |
| #2 | 'non-alcoholic fatty liver':ab,ti OR nafld:ab,ti OR nash:ab,ti OR 'nonalcoholic fatty liver*':ab,ti OR 'nonalcoholic steatohepatiti*':ab,ti OR 'non alcoholic hepat* steatosis':ab,ti OR 'non alcoholic hepatosteatosis':ab,ti OR 'non alcoholic liver steatosis':ab,ti OR 'non alcoholic steatotic hepatopathy':ab,ti OR 'non-alcoholic fld':ab,ti OR 'nonalcoholic fld':ab,ti OR 'nonalcoholic hepatic steatosis':ab,ti OR 'nonalcoholic hepatosteatosis':ab,ti OR 'nonalcoholic liver steatosis':ab,ti | 63339 | 2-Mar-23 |
| #1 | 'nonalcoholic fatty liver'/exp | 63792 | 2-Mar-23 |

| 1. **The retrieval method of Web of science** | | |
| --- | --- | --- |
| NO. | Search mode | Results |
| 14 | (((((((((((((((((((((((((((((TI=(Non-alcoholic Fatty Liver Disease)) OR TI=(non-alcoholic fatty liver)) OR TI=(NAFLD)) OR TI=(NASH)) OR TI=(Nonalcoholic Fatty Liver*)) OR TI=(Nonalcoholic Steatohepatiti*)) OR TI=(non alcoholic hepat* steatosis)) OR TI=(non alcoholic hepatosteatosis)) OR TI=(non alcoholic liver steatosis)) OR TI=(non alcoholic steatotic hepatopathy)) OR TI=(non-alcoholic FLD)) OR TI=(nonalcoholic FLD)) OR TI=(nonalcoholic hepatic steatosis)) OR TI=(nonalcoholic hepatosteatosis)) OR TI=(nonalcoholic liver steatosis)) OR AB=(Non-alcoholic Fatty Liver Disease)) OR AB=(non-alcoholic fatty liver)) OR AB=(NAFLD)) OR AB=(NASH)) OR AB=(Nonalcoholic Fatty Liver*)) OR AB=(Nonalcoholic Steatohepatiti*)) OR AB=(non alcoholic hepat* steatosis)) OR AB=(non alcoholic hepatosteatosis)) OR AB=(non alcoholic liver steatosis)) OR AB=(non alcoholic steatotic hepatopathy)) OR AB=(non-alcoholic FLD)) OR AB=(nonalcoholic FLD)) OR AB=(nonalcoholic hepatic steatosis)) OR AB=(nonalcoholic hepatosteatosis)) OR AB=(nonalcoholic liver steatosis) | 96,851 |
| 24 | (((((((((((((((((((((((((((((((((((((((((((((((((((((((((((((((((((((((((((((((((((((((((((((((((((((((((((((((((((((((((((((TI=(vegetable*)) OR TI=(fruit*)) OR TI=(diet*)) OR TI=(nutrition)) OR TI=(Potato*)) OR TI=(manioc)) OR TI=(cassava)) OR TI=(taro)) OR TI=(carrot*)) OR TI=(beet*)) OR TI=(parsnip*)) OR TI=(turnip*)) OR TI=(spinach)) OR TI=(lettuce)) OR TI=(cabbage*)) OR TI=(bok choy)) OR TI=(broccoli)) OR TI=(watercress)) OR TI=(allium)) OR TI=(onion*)) OR TI=(garlic)) OR TI=(leek*)) OR TI=(Apple*)) OR TI=(banana*)) OR TI=(berries)) OR TI=(berry)) OR TI=(grape*)) OR TI=(citrus)) OR TI=(orange*)) OR TI=(grapefruit*)) OR TI=(lemon*)) OR TI=(lime*)) OR TI=(apricot*)) OR TI=(endive*)) OR TI=(greens)) OR TI=(romaine)) OR TI=(brussels sprout*)) OR TI=(cauliflower)) OR TI=(kohlrabi)) OR TI=(collard*)) OR TI=(kale)) OR TI=(rutabaga*)) OR TI=(Umbelliferous)) OR TI=(Celery)) OR TI=(parsley)) OR TI=(fennel)) OR TI=(shallot* )) OR TI=(chive)) OR TI=(eggplant)) OR TI=(tomato*)) OR TI=(Gourd*)) OR TI=(pumpkin*)) OR TI=(squash)) OR TI=(cucumber*)) OR TI=(muskmelon*)) OR TI=(watermelon*)) OR TI=(melon*)) OR TI=(Mustard)) OR TI=(solanaceous)) OR TI=(cucurbitaceous)) OR TI=(sage)) OR TI=(dill)) OR TI=(mint)) OR TI=(Cruciferous)) OR AB=(vegetable*)) OR AB=(fruit*)) OR AB=(diet*)) OR AB=(nutrition)) OR AB=(Potato*)) OR AB=(man | 5,126,076 |
| 25 | #24 AND #14 | 15,258 |
| 27 | (TI=(mouse)) OR AB=(mouse) | 2,121,128 |
| 30 | #25 NOT #27 | 8,916 |

| 1. **The retrieval method of the Cochrane Library** | | |
| --- | --- | --- |
| Search Name: |  |  |
| Date Run: | 02/03/2023 20:42:54 |  |
| Comment: |  |  |
|  |  |  |
| ID | Search | Hits |
| #1 | MeSH descriptor: [Non-alcoholic Fatty Liver Disease] explode all trees | 1532 |
| #2 | (non-alcoholic fatty liver or NAFLD or NASH or Nonalcoholic Fatty Liver* or Nonalcoholic Steatohepatiti* or non alcoholic hepat* steatosis or non alcoholic hepatosteatosis or non alcoholic liver steatosis or non alcoholic steatotic hepatopathy or non-alcoholic FLD or nonalcoholic FLD or nonalcoholic hepatic steatosis or nonalcoholic hepatosteatosis or nonalcoholic liver steatosis):ti,ab,kw | 4968 |
| #3 | #1 or #2 | 4968 |
| #4 | vegetable* or diet* or nutrition or fruit* or Potato* or manioc or cassava or taro or carrot* or beet* or parsnip* or turnip* or spinach or lettuce or cabbage* or bok choy or broccoli or watercress or allium or onion* or garlic or leek* or Apple* or banana* or berries or berry or grape* or citrus or orange* or grapefruit* or lemon* or lime* or apricot* or endive* or greens or romaine or brussels sprout* or cauliflower or kohlrabi or collard* or kale or rutabaga* or Umbelliferous or Celery or parsley or fennel or shallot* or chive or eggplant or tomato* or Gourd* or pumpkin* or squash or cucumber* or muskmelon* or watermelon* or melon* or Mustard or solanaceous or cucurbitaceous or sage or dill or mint or Cruciferous | 156972 |
| #5 | #3 and #4 | 1626 |
