# Supplementary figures and images for: Fruit and vegetable intake and the risk of non-alcoholic fatty liver disease: a meta-analysis of observational studies
Source: Front Nutr. 2024 Jun 21;11:1398184. doi: 10.3389/fnut.2024.1398184 (PMC11224539; doi:10.3389/fnut.2024.1398184)

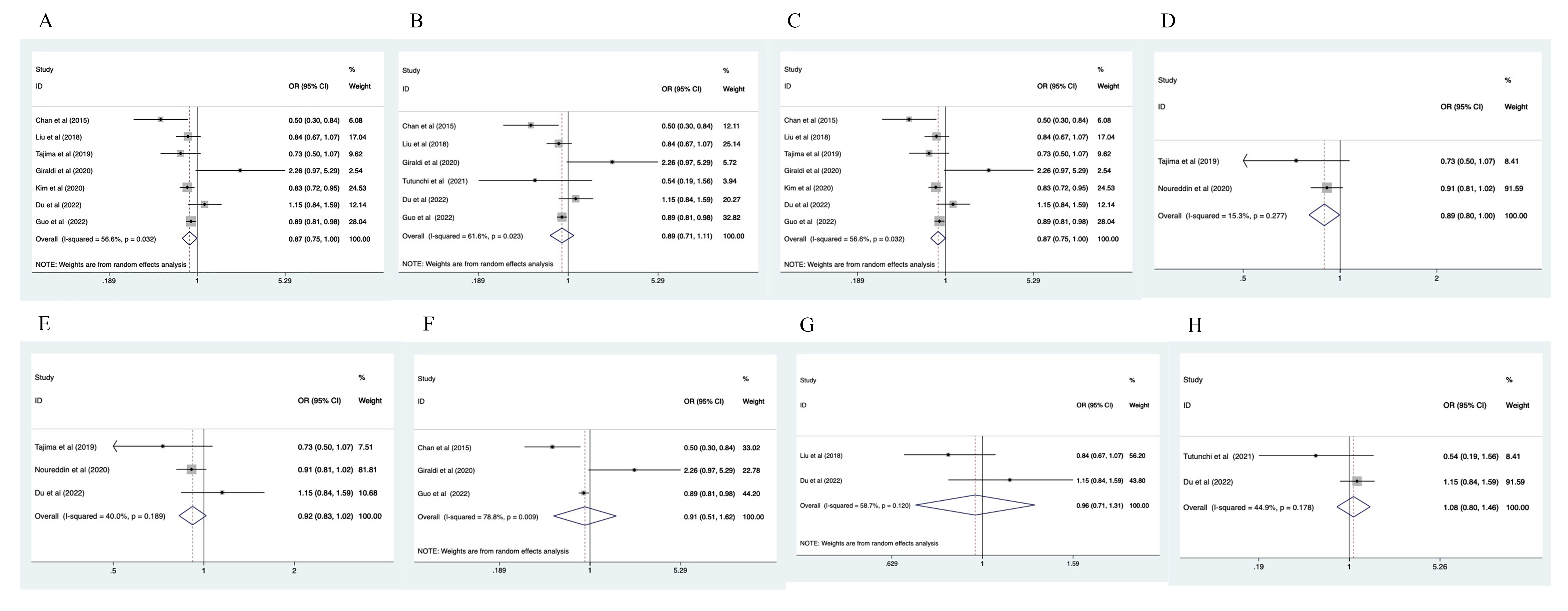

Supplement: Appendix 1 — The complete retrieval formula. [file Data_Sheet_1.ZIP › Supplementary materials/Supplementary figures/fig S6.tif]

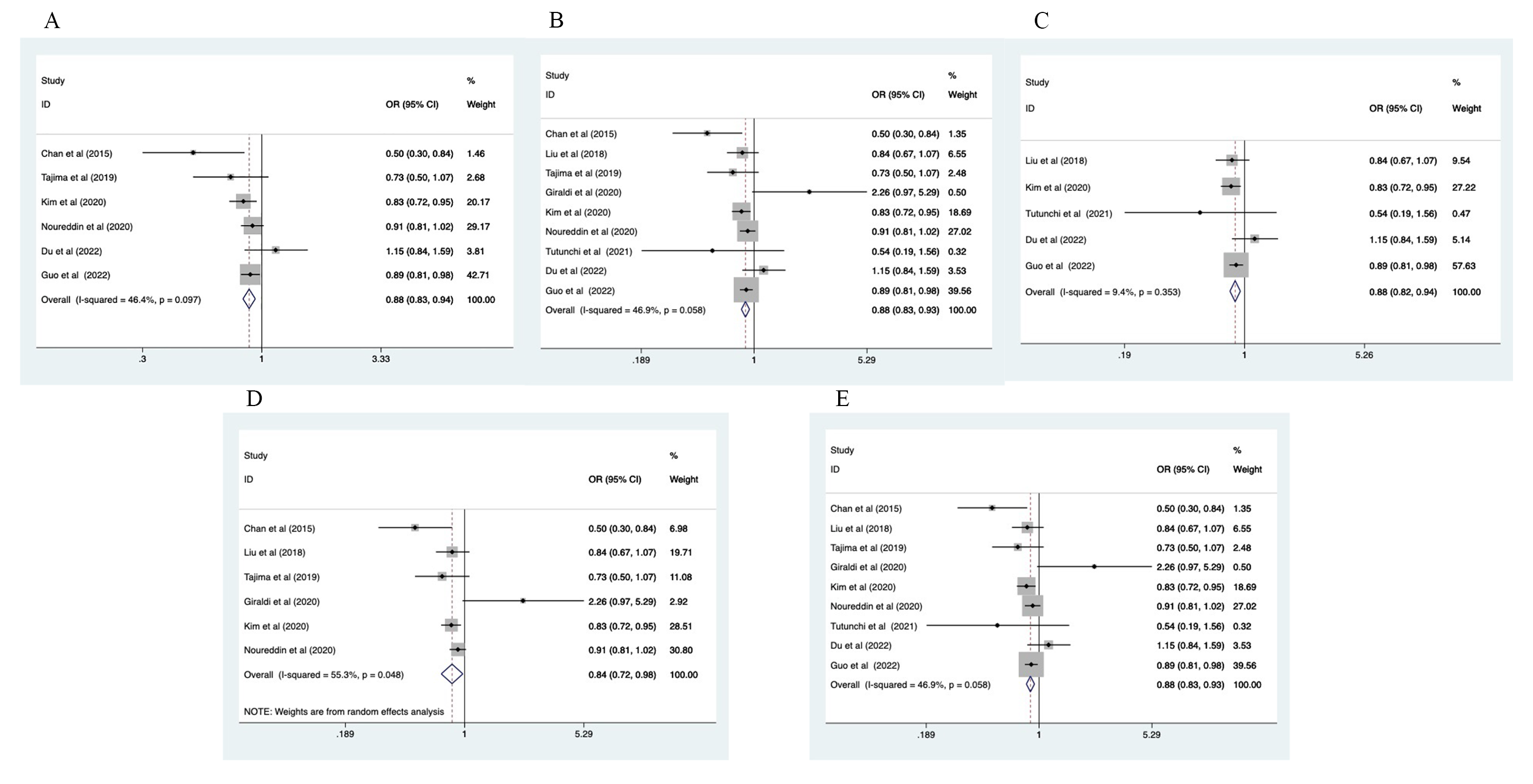

Supplement: Appendix 1 — The complete retrieval formula. [file Data_Sheet_1.ZIP › Supplementary materials/Supplementary figures/fig S5.tif]

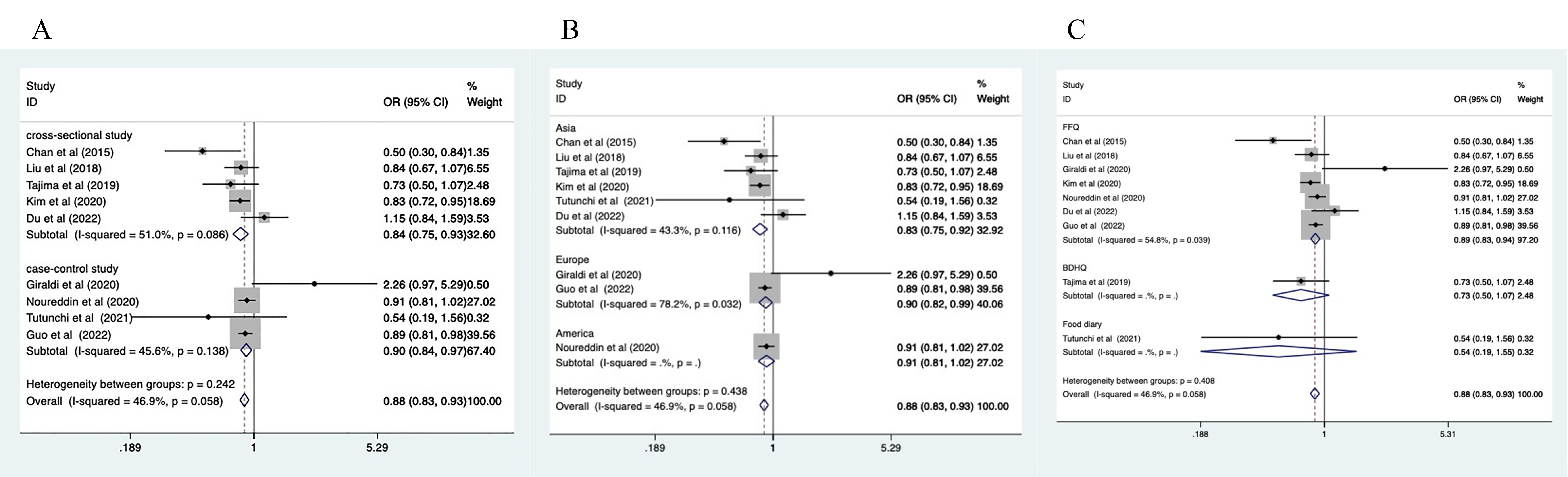

Supplement: Appendix 1 — The complete retrieval formula. [file Data_Sheet_1.ZIP › Supplementary materials/Supplementary figures/fig S4.tif]

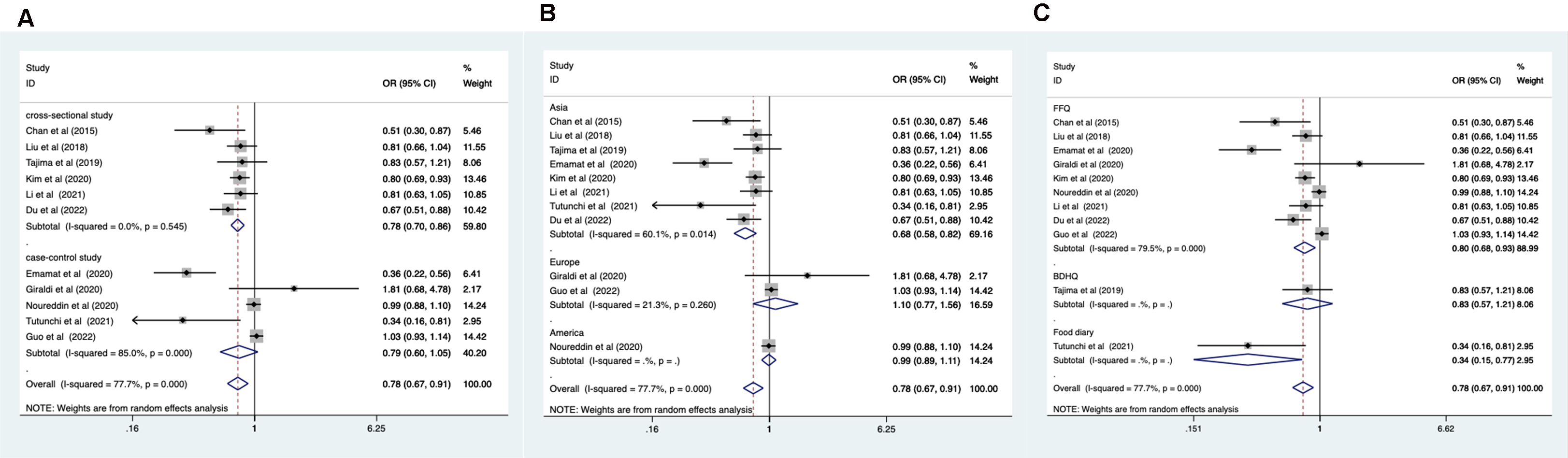

Supplement: Appendix 1 — The complete retrieval formula. [file Data_Sheet_1.ZIP › Supplementary materials/Supplementary figures/fig S1.tif]

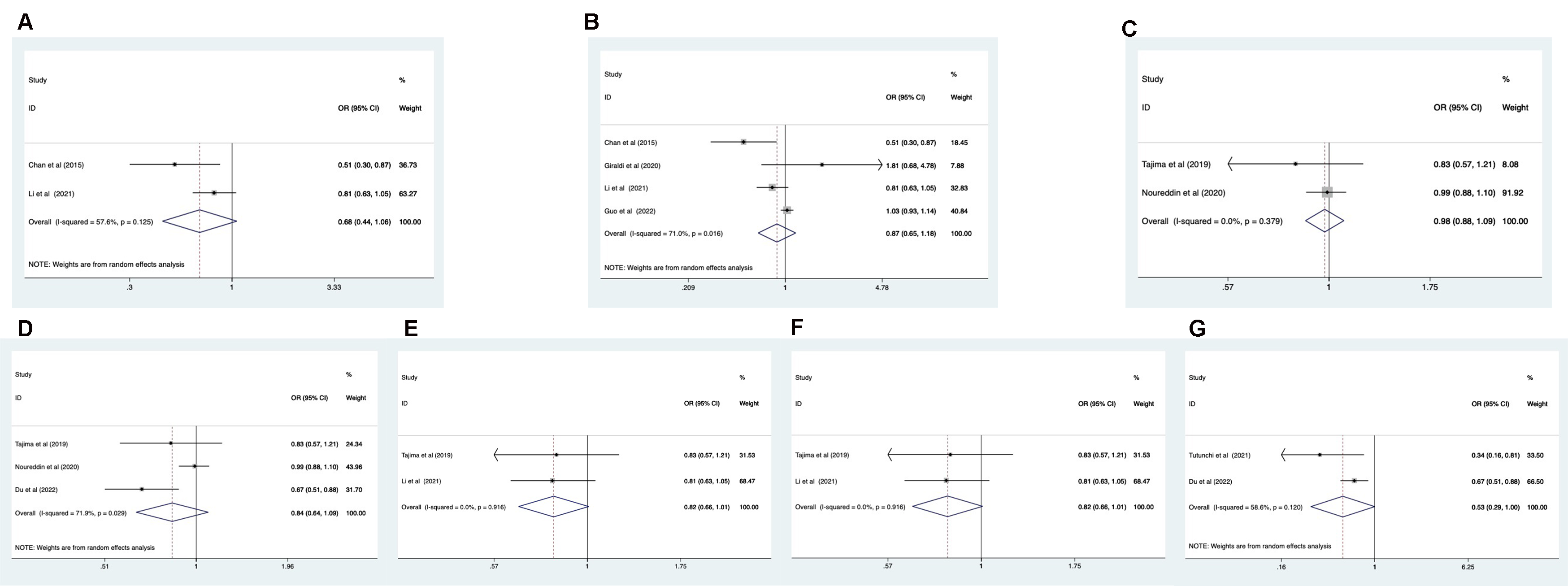

Supplement: Appendix 1 — The complete retrieval formula. [file Data_Sheet_1.ZIP › Supplementary materials/Supplementary figures/fig S3.tif]

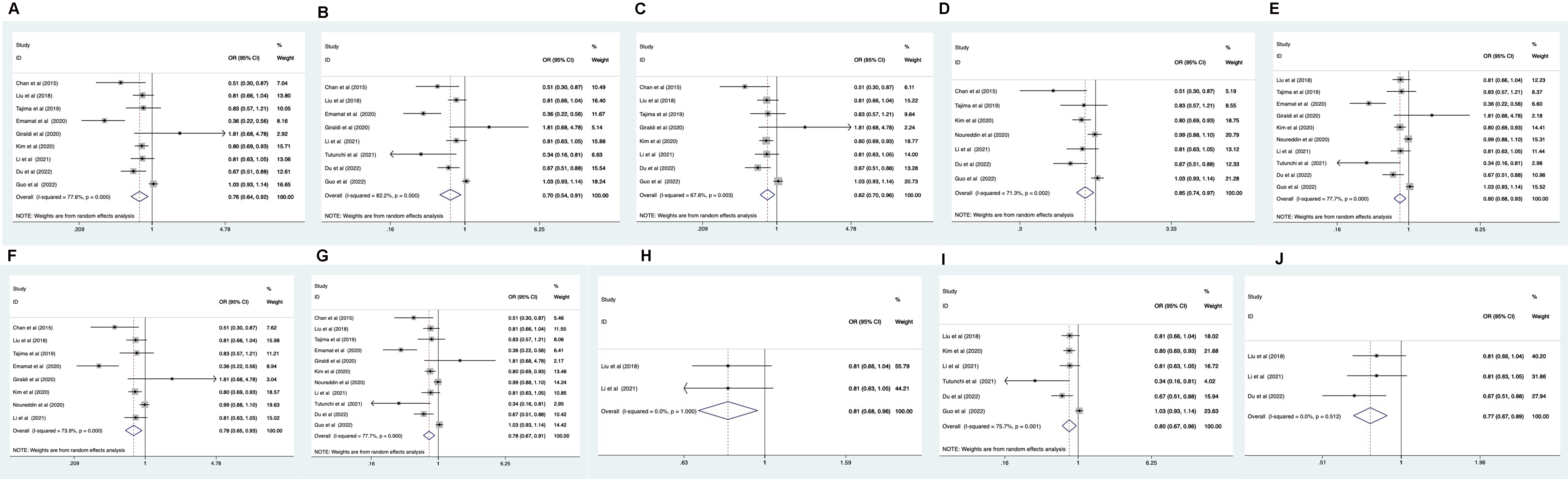

Supplement: Appendix 1 — The complete retrieval formula. [file Data_Sheet_1.ZIP › Supplementary materials/Supplementary figures/fig S2.tif]
